# Sources of particle number concentration and noise near London Gatwick Airport

**DOI:** 10.1016/j.envint.2022.107092

**Published:** 2022-03

**Authors:** Anja H. Tremper, Calvin Jephcote, John Gulliver, Leon Hibbs, David C. Green, Anna Font, Max Priestman, Anna L. Hansell, Gary W. Fuller

**Affiliations:** a MRC Centre for Environment and Health, Environmental Research Group, Imperial College London, London, UK; b Centre for Environmental Health and Sustainability, University of Leicester, Leicester, UK; c Environmental Health, Reigate & Banstead Borough Council, Town Hall, Reigate, Surrey, UK

**Keywords:** Source apportionment, Ultrafine particles, Particle number size distributions, Traffic, Airport emissions, Noise

## Abstract

There is increasing evidence of potential health impacts from both aircraft noise and aircraft-associated ultrafine particles (UFP). Measurements of noise and UFP are however scarce near airports and so their variability and relationship are not well understood. Particle number size distributions and noise levels were measured at two locations near Gatwick airport (UK) in 2018–19 with the aim to characterize particle number concentrations (PNC) and link PNC sources, especially UFP, with noise. Positive Matrix Factorization was used on particle number size distribution to identify these sources. Mean PNC (7500–12,000 p cm^−3^) were similar to those measured close to a highly trafficked road in central London. Peak PNC (94,000 p cm^−3^) were highest at the site closer to the runway. The airport source factor contributed 17% to the PNC at both sites and the concentrations were greatest when the respective sites were downwind of the runway. However, the main source of PNC was associated with traffic emissions. At both sites noise levels were above the recommendations by the WHO (World Health Organisation). Regression models of identified UFP sources and noise suggested that the largest source of noise (LAeq-1hr) above background was associated with sources of fresh traffic and urban UFP depending on the site. Noise and UFP correlations were moderate to low suggesting that UFP are unlikely to be an important confounder in epidemiological studies of aircraft noise and health. Correlations between UFP and noise were affected by meteorological factors, which need to be considered in studies of short-term associations between aircraft noise and health.

## Highlights

• Particle size distributions and noise levels were measured at two locations near Gatwick airport in 2018–19.
• Peak particle number concentrations (PNC) were highest at the site closer to the runway.
• Source apportionment identified six factors at each site with the airport source factor contributing 17%.
• The largest source of noise above background was associated with sources of fresh traffic and urban particles depending on the site.
• PNC is unlikely to be an important confounder in epidemiological studies of aircraft noise and health near airports.

## Graphical abstract

**Figure:**
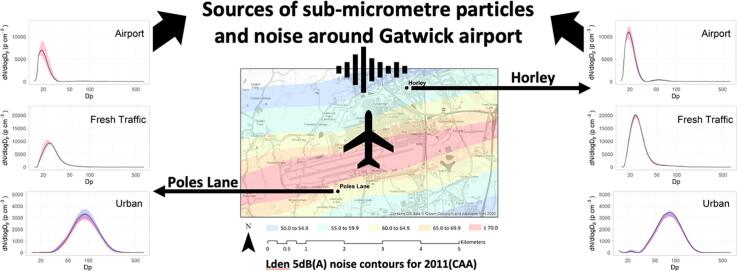

## 1 Introduction

There is increasing evidence of potential health impacts from both aircraft noise and aircraft-associated air pollution exposure to local communities around airports (Hansell et al., 2013, Lammers et al., 2020). Air pollution from aircraft engines and auxiliary power units include CO, CO_2_, H_2_O, SO_2_, NO_X_ (NO + NO_2_), a range of hydrocarbons (HC) and particulate matter (PM) (Barrett et al. 2010). These emissions can lead to air pollution exposure that extend beyond airport perimeters. For instance, Carslaw et al. (2006) detected NO_2_ concentrations from Heathrow Airport in communities 3 km away from Heathrow Airport.

Whilst exposure to regulated pollutants is reasonably well characterised, aircraft also give rise to high number concentrations of ultrafine particles (UFP) that are poorly understood and characterised. Airports challenge aircraft engines with diverse operating conditions from idle to taxi to full thrust at take-off, thereby causing large variations in particle number emissions. High, variable and poorly controlled sulphur content of aviation fuel is also a key factor in UFP emissions (Miller et al., 2009, Schumann et al., 2002). Measurements of NO_2_ and PM mass concentration have been found to be poor surrogates for airport UFP due to multiple sources in urban areas (Herndon et al., 2005, Stacey, 2019) and direct measurements are therefore needed to understand community exposure.

Initial evidence of airports as a large source of UFP arose from measurements of 100 flights at John F. Kennedy International Airport (New York City) and Logan Airport (Boston) in the United States (Herndon et al. 2005). Westerdahl et al. (2008) found impacts on the surrounding communities with surprisingly high particle number concentrations (∼5 × 104 p cm^−3^) 500 m downwind of Los Angeles International Airport (LAX) and that the airport contributed to ambient particle number up to 900 m downwind of a runway. Zhu et al. (2011) also found particle number concentration that exceeded background concentrations at ∼600 m downwind from LAX. Increased airport UFP concentrations have also been measured in the indoor environment, specifically in a home approximately 1.3 km from Boston’s Logan Airport (Hudda et al. 2020).

More recent measurement campaigns have investigated UFP concentrations at greater distances from airports. Hudda and Fruin (2016) detected ultrafine particles from LAX (Los Angeles) suburbs 18 km downwind of the airport. In the Dutch countryside, Keuken et al. (2015) measured elevated particle number concentrations around 40 km downwind from Schiphol Airport. (Harrison et al. 2019) detected elevated particle number concentration in central London when the wind arrived from the direction of Heathrow airport, 22 km away.

To get a better insight into the contribution airports have on the overall UFP concentration it is important to distinguish different UFP sources. Source apportionment using receptor models can provide this insight and the observed number size concentrations can be utilised (Ogulei et al., 2006, Ogulei et al., 2007, Beddows et al., 2015). Masiol et al. (2017) used positive matrix factorisation to investigates the sources of sub-micrometre particles near London Heathrow airport, and found that airport sources substantially contributed to UFP. Source apportionment of particle number and size measurements in the centres of Barcelona, Helsinki, London, and Zurich by Rivas et al. (2020) detected UFP from each city’s airport providing evidence of large population exposure even at greater distances to the airports.

Previous reviews by Health Effects Institute (2), Ohlwein et al. (2019) and more recent commentaries (https://efca.net/files/WHITE%20PAPER-UFP%20evidence%20for%20policy%20makers%20(25%20OCT).pdf) have provided suggestive evidence of adverse effects of short-term and long-term exposure to UFP on mortality, pulmonary/systemic inflammation and cardiovascular outcomes. Systematic reviews of evidence on aircraft noise have also found associations with cardiovascular outcomes (van Kempen et al. 2018), but it has been suggested that associations between aircraft noise and cardiovascular outcomes (e.g., Hansell et al, 2013) may have been confounded by UFP exposure (Corbin 2013).

Reviews considering traffic noise and air pollution have concluded that these have independent effects (Stansfeld, 2015, Tetreault et al., 2013). Most studies have considered road noise as the noise source together with routinely monitored air pollutants. Almost no studies have considered co-exposure to UFP (which are not routinely monitored). While there have been a small number of assessments of co-exposure to UFP from aircraft and aircraft noise in occupational studies of airport workers (Buonanno et al., 2012, Marcias et al., 2019, Lecca et al., 2021), we are not aware of such studies in community settings.

The aim of this study was to identify sources and their contributions of sub-micrometre particles to the concentration of particle number concentrations around UK’s second largest airport. This was to provide information on whether and under what circumstances UFP might be correlated with noise exposure and therefore might need to be taken into consideration in studies investigating associations between aircraft noise and health outcomes.

## 2 Methodology

### 2.1 Monitoring periods and location

Measurements were taken close to London Gatwick airport (LGW), which lies 40 km to the south of London. The airport is the UK’s second busiest airport (46.4 million passengers for the 12 months before 31st March 2019) after London Heathrow (80.1 million passengers in 2018) (Gatwick Airport Limited, 2019, Heathrow (SP) Limited, 2019). It has one runway, which accommodated 284,000 aircraft movements in 2018; take-offs and landings to the east or west of the airport were dependent on the wind direction.

Gatwick is situated in a predominantly rural area with the town of Horley (population ∼25,000) to the north/north-east and Crawley (population ∼112,000), the largest town in the area, to the south. The most heavily trafficked road is the M23 motorway runs north-south 2 km east of the airport, and the A23, which runs north-south close to the airport and is the main airport feeder road.

**Fig. 1:**
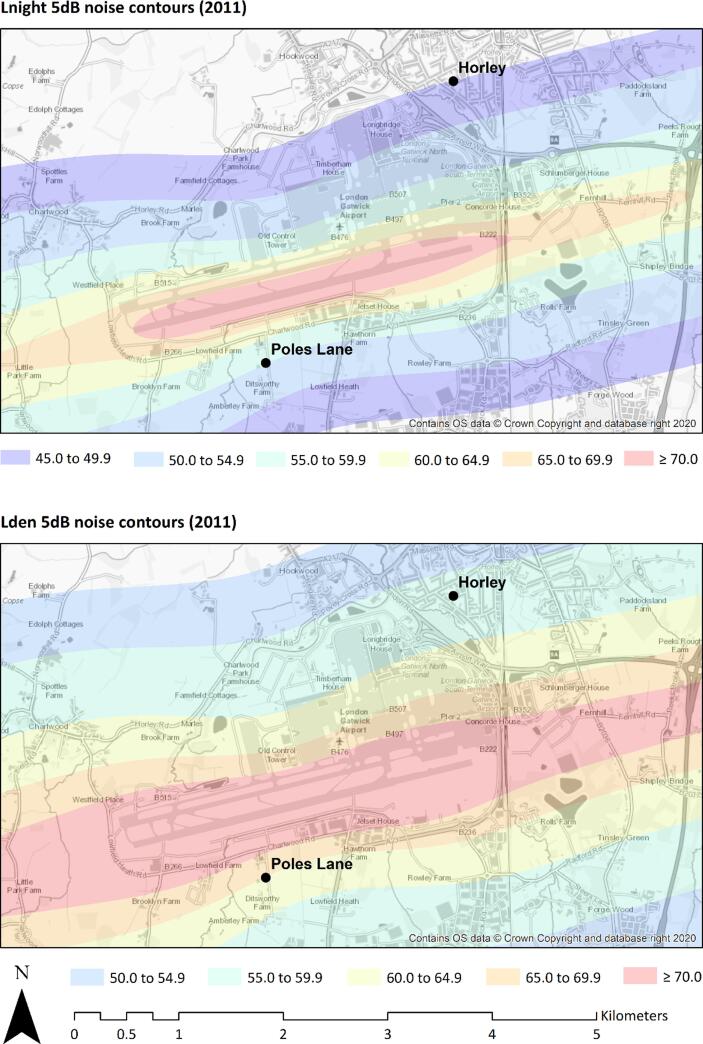
Civil Aviation Authority (CAA) modelled aircraft noise contours around Gatwick airport for night-time (Lnight) and weighted daily average (Lden) noise levels, and monitoring site locations: Horley and Poles Lane.

One scanning mobility particle sizer was installed sequentially at two existing air quality monitoring stations in 2018. An overview of the two monitoring campaigns is given in Table 1. The locations of these monitoring stations were historically selected to be on opposite sides of the airport along the approximate prevailing wind direction. The Horley site is classified as a suburban industrial site, located 1.6 km from the far eastern end of the runway and around 0.6 km from the airport perimeter. The site is 0.8 km from north terminal and 0.9 km from south terminal buildings. The monitoring campaign lasted 12 weeks starting 20 July 2018. The Poles Lane site is in a rural setting, about 0.6 km from centre of the runway, 0.3 km from the airport perimeter, 2.2 km from the north terminal and 2.6 km from the south terminal. The monitoring campaign lasted 14 weeks starting 17/10/2018. Both sites were within an area that exceeds noise level recommendations by the World Health Organisation (WHO) of L_den_ (Day-evening-night-weighted sound pressure level) < 45 dB and L_night_ (Equivalent continuous sound pressure level when the reference time interval is the night < 40 dB (WHO 2018). The locations of the monitoring sites are shown alongside the aircraft noise contours as modelled by Civil Aviation Authority are shown in Fig. 1.

**Table 1:** Overview of sampling campaigns.

Site Name	Horley	Poles Lane
Lat / Long	51.166/−0.168	51.142/−0.194
Start Date	20 July 2018	17 October 2018
End Date	15 October 2018	22 January 2019
Site Type	urban background	rural
Measured	PNC, PNSD, NO, NO2, NOX, BC880, BC370, PM10, PM10Vol, Noise	PNC, PNSD, NO, NO2, NOX, BC880, BC370, PM10Vol*, Noise
	*PM10vol fraction measurements taken from Horley

Both sites comply with the WHO air quality guideline (WHO 2006) for NO_2_ in 2018 with annual means < 40 µg m^−3^ and less than 18 instances with hourly mean concentrations exceeding 200 µgm^−3^. Horley had a higher 2018 annual mean (20 µgm^−3^) than Poles Lane (16 µgm^−3^). At Horley the WHO air quality guideline for PM_10_ (<20 µg m^−3^ annual mean and 50 µg m^−3^ as a 24 h mean, not to be exceeded more than 35 times a year) were also achieved; PM_10_ is not routinely measured at Poles Lane.

Hourly meteorological records from Gatwick Airport were extracted from the National Oceanic and Atmospheric Administration (NOAA) Integrated Surface Database (https://www.ncdc.noaa.gov/isd) using the *worldmet* R package (Carslaw 2020).

For the evaluation of pollution concentrations, the air quality data from the two measurement sites were compared to three national network sites (https://uk-air.defra.gov.uk/networks/network-info?view=particle), namely London Marylebone Road (roadside site), London North Kensington (urban background site) and Chilbolton (rural site). These sites were chosen as they are well characterised observatories with all instruments of interest and were used in many previous publications for comparison (Stacey et al., 2020, Rivas et al., 2020, Bohnenstengel et al., 2015, Beccaceci et al., 2016).

### 2.2 Instrumentation

During the sampling campaigns, the particle size distribution was measured in 107 size bins (14.6–661.2 nm) using a Scanning Mobility Particle Sizer (SMPS), consisting of an electrostatic classifier (Model 3080, TSI Inc., USA), differential Mobility Analyser (DMA Model 3081, TSI, USA) and a Condensation Particle Counter (Model 3775, TSI Inc., USA). The sample air was dried to below 45% relative humidity (RH) as recommended by the EUSAAR protocol (Wiedensohler et al. 2012) using a membrane dryer (MD-700-24F-1, Perma Pure LLC, USA). The analyser was operated using the AIM9 software with multiple charge correction and diffusion loss correction enabled. Data measured each fifteen-minutes with 6 scans per sample were averaged to hourly values. The sum of concentrations in each bin was also reported as particle number concentration (PCN).

The systems were visited every two to three weeks for leak checks and to ensure flows remained ±10 % of the set rate. During these visits the impactor and sample inlets were cleaned in an ultrasonic bath. The instrument was fully serviced and calibrated before deployment. This included full recalibration of flows, leak checks, linearity checks and aerosol tests to confirm size accuracy and resolution. These were all within the manufacturer’s specification.

Dual wavelength aethalometers (AE22, Magee Scientific, USA) were also installed at both sites to measure black carbon (BC_880_) and the UV component (BC_370_) during the campaigns (Font and Fuller, 2016, Butterfield et al., 2016).

Each location was equipped with a chemiluminsecent NO_X_ analyser (Horley: Model ML9841B, Teledyne Monitor Labs, USA; Poles Lane: Model T200, Teledyne Advanced Pollution Instrumentation, USA) to measure nitrogen oxide (NO), nitrogen dioxide (NO_2_) and total oxides of nitrogen (NO_X_). Additionally, PM_10_ was measured using a TEOM-FDMS (1400AB/8500CB, Thermo Fisher Scientific, USA). The volatile measurement data (PM_10vol_) from the TEOM-FDMS instrument at Horley was used for data analysis at both sites as the volatile component is representative of secondary inorganic compounds, such as ammonium nitrate, in an area up to 200 km (Green et al. 2009). PM and gaseous monitoring quality assurance and control followed European Union Standards (Directive 2008/50/EC).

For the noise measurements at both sites a NoiseMote (Envirowatch Ltd, UK) was used. The instruments measure noise through an A-weighted filter, which covers a frequency range of 20 Hz to 20 kHz, and is designed to reflect the response of the human ear to noise. The MOTE instruments captured noise measurements at 1-minute intervals by sampling the noise 8 times per second. These 1-minute measurements were then summarised to equivalent continuous sound levels at hourly intervals (L_Aeq-1hr_). LAeq is the abbreviation for: [A]-weighted, [eq]uivalent continuous sound level. The NoiseMotes have a 50 dB(A) range that can be adjusted to lie between the lower bound of 40 dB(A) and the upper bound of 125 dB(A). The device was tested in an anechoic environment and the measurements were always within 2.2 dB(A) of precision measurements in this range, meeting Class 2 requirements.

### 2.3 Statistical analysis

The statistical analysis was conducted in the R programming language (libraries used include e.g., Openair, Worldmet (Carslaw and Ropkins, 2012, Carslaw, 2020). Levels of multiple collinearity were measured with ‘usdm’ (Naimi et al. 2014), and the polar plots were created in ‘openair’.

In line with British Medical Journal suggestions (BMJ 2021), correlations (R^2^) were termed as very weak (|0-0.19|), weak (|0.2–0.39|), moderate (|0.4–0.59|), strong (|0.60–0.79|) and very strong (|0.8–1.0|).

Positive matrix factorisation was performed using the United States Environmental Protection Agency (US EPA) PMF5 model (Norris et al. 2014) is described below.

#### 2.3.1 Positive matrix factorisation (PMF)

Positive matrix factorisation (PMF) is a multivariate factor analysis tool, which is widely used to quantify the contribution of sources to measured concentrations by analysing the measured chemical or size composition at a receptor site; the model is detailed elsewhere (Paatero, 1997, Paatero and Tapper, 1994). Here, PMF was used to identify and apportion different sources to the measured particle size distribution.

The U.S. EPA PMF5 model was utilised to perform PMF analysis (Norris et al., 2014). The model inputs were the hourly particle size distribution and uncertainty estimates calculated according to the methodology established by Ogulei et al. (2007) with variation as described by Rivas et al. (2020); see supplement. The treatment of missing values followed the methodology of Rivas et al. (2020). The model was run separately for each campaign and 3–8 factor solutions were investigated for both sites. The best solutions were identified and interpreted using the following criteria:

- the scaled residuals should be approximately randomly distributed between −3 and 3
- the object function Q (sum of scaled residuals) should be closest to but higher than the theoretical value of 1
- correlation between the time series of different factors should be low as this might otherwise indicate splitting of factors
- correlation between factors and other pollutants were considered for factor interpretation
- the factor solutions should have physically meaningful profiles and temporal behaviour

Further, an a-priori condition that the solution should have a factor indicative of the airport emissions was included in the criteria as this was important for the evaluation of the airport associated particle pollution in respect to airport noise. This factor was assumed to be in the nucleation mode (14–30 nm) as previous studies showed that this is the size range predominantly associated with airports (Yu et al., 2019, Masiol et al., 2017, Hudda and Fruin, 2016, Stacey, 2019) and expected to have a clear relationship with airport activity. However, no constraints were introduced to the model to identify this factor.

The model result uncertainties were calculated using bootstrap (BS, n = 100) and displacement (DISP.) methods; further model analysis details please see supplement 2.2.

#### 2.3.2 Exploratory data analysis (EDA)

A series of Pearson’s R and Spearman’s Rho correlations were calculated between hourly measured noise levels and a single measure of airport activity, meteorology, or a source specific particle number concentration (Table 5).

Pairwise correlation coefficients can range from +1 to −1, indicating either a perfect positive, or a perfect negative relationship where one variable increases while the other decreases: Values of 0 to ±0.19 indicate a ‘very weak’, ±0.20 to ±0.39 indicate a ‘weak’, ±0.40 to ±0.59 indicate a ‘moderate’, ±0.60 to ±0.79 indicate a ‘strong’, and ±0.80 to ±1 indicate a “very strong” relationship between the two variables of interest, where P ≤ 0.05.

Pearson’s R directly measures the linear relation of two variables, whereas Spearman’s Rho tests for non-linear relation on variables that have been ranked and ordered. Differences in magnitude between the two tests indicate if a predominantly linear or non-linear pairwise relationship exists.

To control for the complex influences on noise and understand the interaction between noise and particle number count and respective sources, a series of linear regression models were used (Table 6). Models 1 and 4, for the Horley and Poles Lane campaigns respectively, only included meteorological parameters. Models 3 and 6 included the primary pollution sources, adjusting for the confounding influence of meteorology. In all of these models, the dependent (y) variable is a series of hourly noise measurement that are in decibels (LAeq-1hr). The continuous predictor (x) variables were normalised on a 0–1 scale, to adjust for any disparities in variable size, ensuring that the regression model coefficients (effect sizes) are in proportion with one another. Therefore, the model coefficients are reflective of the maximum influence that a given parameter has on noise, and the size of the coefficient directly indicates the importance of a parameter.

The following meteorological parameters were considered for inclusion in the regression models: air temperature (dry-bulb) measured in degrees Celsius; liquid-precipitation depth measured in millimetres at the time of an observation and for the previous five hours; relative humidity as a percentage; and wind speed measured in metres-per-second. Wind direction is included as a categorical variable, where the effect of winds from the NE (0–89°), SE (90–179°), and NW (270–359°) are individually compared to the prevailing wind direction in England (SW = 180–269°).

Meteorological and source specific parameters were only included in the models, if they did not have a high pairwise correlation with any of the other explanatory variables entering the model (R > 0.6 or R < −0.6). Collinearity causes instability in parameter estimation in regression-type models. Thus, relative humidity was omitted from model 1, due to its high correlation with air temperature and the fresh traffic factor was omitted from model 6 because of its high correlation with the airport factor.

A Variance inflation factor (VIF) test was also run to check for multiple collinearities between more than two predictor variables. The test is based on the square of the multiple correlation coefficient resulting from regressing a predictor variable against all other predictor variables. VIF scores below two were recorded in models 1–6, which indicates that no multicollinearity issues exist between the candidate explanatory variables in any of our models (Schuenemeyer and Drew 2011).

Two regression statistics were also included in the analysis. Firstly, adjusted r-squares were used to show the fit of the model to the data (i.e., determines the proportion of variance in the dependent variable that can be explained by the independent variables). Secondly, Lilliefors Kolmogorov-Smirnov Residual Normality Test (with 10,000 bootstrap replications) was used to check the distribution of residuals, where a p-value > 0.05, confirms that the residuals are normally distributed.

## 3 Results and discussion

### 3.1 Overview

The two sampling campaigns were carried out in different seasons and thus the ambient conditions differed substantially. Wind roses for both sites/seasons are given in the supplement (Fig. S2) and show that at Horley the campaign was dominated by south-westly wind (40–45% of the time) with wind speeds up to 9.8 m s^−1^. During the campaign in Poles Lane, the prevailing wind direction was also from the south west (25–30% of the time) with wind speeds up to 10.3 m s^−1^ but the wind direction varied more than during the campaign in Horley. Mean air temperature during the campaign at Horley were 16.5 °C and the maximum/minimum temperatures were 33 and 2 °C, respectively. During the campaign in Poles Lane, the mean temperature was 7.7 °C with a maximum/minimum temperature of 19 and −5 °C, respectively. The diurnal air temperature profiles are shown in Fig. S2.

#### 3.1.1 Horley campaign

An overview of the campaign measurements is provided in Table 2. During the sampling campaign in Horley the mean (median) particle count concentration was 11,745 (9016) p cm^−3^. Total PNC as measured by an SMPS has a higher uncertainty than those measured by a CPC of similar quality (Beccaceci et al., (2013)). A comparison between SMPS and CPC was not available in this study but Beccaceci et al., (2013) and Harrison et al. (2019) reported higher concentrations measured with CPC than SMPS, mainly due to a wider size range. Compared with concentrations around London’s Heathrow airport, the concentrations of SMPS PNC found in this study were lower than the average concentrations measured by SMPS during the warm season in 2014 at Harlington, at a site 1.2 km away from Heathrow (19,000 p cm^−3^, (Masiol et al. 2017)) but higher than those measured at Oaks Road and LHR2, two locations within 1 km of London Heathrow Airport in 2016 (mean: 8911–7408 p cm^−3^; 4756–3948 p cm^−3^) (Stacey et al. 2020).

**Table 2:** Summary of hourly pollutant concentration during measurement campaigns.

Hourly Concentrations at Horley									
	PNC Count	NOX	NO2	NO	BC880	BC370	PM10	PM10VOL	LAeq-1hr
	(p cm^−3^)	(µg m^−3^)							(dB)
Min.	410	2.6	0.16	0.66	−0.08	0.0048	−1	−2.8	49
1st Qu.	5400	14	9.2	2.3	0.33	0.37	7.4	1.5	53
Median	9000	23	16	3.9	0.57	0.61	9.7	2.5	56
Mean	12,000	29	18	7.5	0.75	0.9	11	2.6	56
3rd Qu.	15,000	35	24	6.9	0.94	1.1	13	3.6	59
Max.	91,000	270	83	150	7.3	9.5	44	11	74
Data capture (%)	97	94	94	94	100	100	99	99	100

Hourly Concentrations at Poles Lane									

	PNC Count	NOX	NO2	NO	BC880	BC370		PM10VOL*	LAeq-1hr
	(p cm^−3^)	(µg m^−3^)							(dB)

Min.	170	1.2	1	−0.19	−0.082	−0.03		−2.7	48
1st Qu.	2000	8.4	7.5	0.4	0.19	0.26		1.6	54
Median	4300	18	15	0.86	0.43	0.67		2.9	58
Mean	7500	28	19	5.7	0.64	1		3.2	58
3rd Qu.	9000	36	28	4.2	0.86	1.4		4.4	62
Max.	94,000	280	80	140	5.2	7.5		14	79
Data capture (%)	97	100	100	100	89	89		99	92

*measured at Horley monitoring station									

**Fig. 2:**
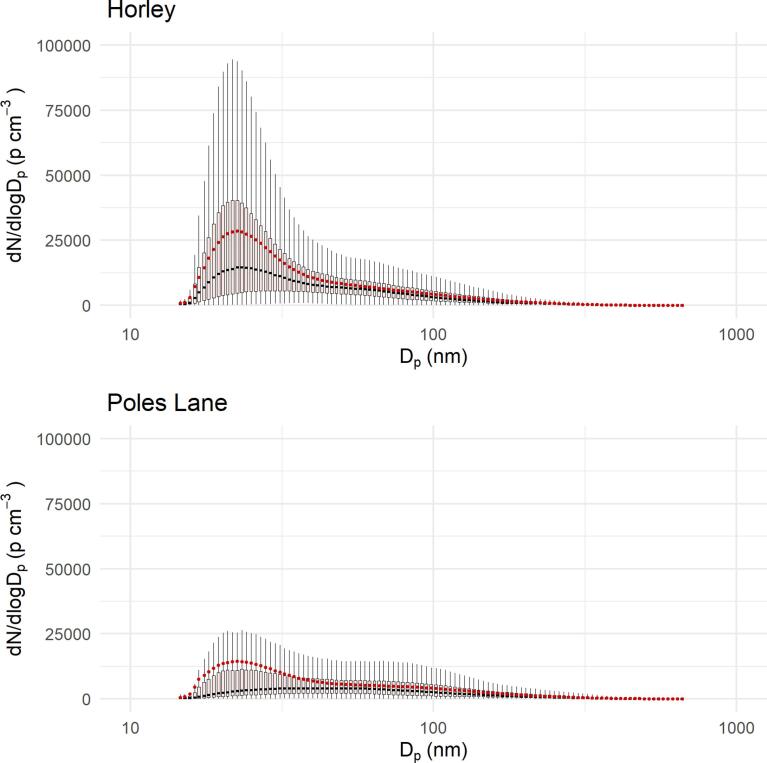
Box and whisker plot with mean (red) and median (black) of PNC size distribution during the sampling campaigns in Horley (top) and Poles Lane (bottom). (For interpretation of the references to colour in this figure legend, the reader is referred to the web version of this article.)

Fig. 2 summarises the particle size distribution; mean and median size distributions were dominated by the nucleation mode (14–30 nm) at Horley. This was in agreement with studies at other airports (Hudda and Fruin, 2016, Keuken et al., 2015, Riley et al., 2016, Masiol et al., 2017).

National network measurements for the same period as the Horley campaign with overlapping times (see Fig. S3) showed that the London roadside site measured similar concentrations (mean: 12,300 p cm^−3^, median: 11,984 p cm^−3^) compared with Horley with a mean of 11,745 p cm^−3^ and a median of 9016 p cm^−3^. However, Horley showed higher concentrations than the London background site (mean: 6142 p cm^−3^, median: 4218 p cm^−3^) and the rural site (mean: 3102 p cm^−3^, median: 2463 p cm^−3^). Maximum concentrations were generally highest at Horley which reflects the influence of the airport as the site is downwind of the airport and major roads the majority of the time.

Diurnal profiles of the particle number concentrations (Fig. S4) show that it was influenced by the morning and evening rush hour. The concentrations stayed elevated the majority of the time and only dropped to a minimum in the early morning hours; this was a reflection of the airport activity (diurnal profiles of flight number is given in Fig. S6), associated traffic as well as typical human activity in the urban area.

The mean and median energy averaged noise levels (L_Aeq-1hr_) during the campaign at Horley were 56 dB and the diurnal variation was similar to that of the total flight numbers (See Figs. S5 and S6). During the campaign, the median flight numbers were 46 flights per hour (diurnal variation of flights is given in Fig. S6), with maximum flight numbers per hour of 56 flights.

Concentrations of other pollutants are also given in Table 2. The mean NO_2_ concentrations during the campaign in Horley (17.6 µg m^−3^) was lower than the concentrations found at the London urban background site (North Kensington, 23.7 µg m^−3^) and roadside site (Marylebone Road 88.4 µg m^−3^) measured in the same period but higher than that found at the rural site (Chilbolton, 6.2 µg m^−3^). The PM_10_ concentrations at Horley were higher than that found at a rural site (Chilbolton, 9.3 µg m^−3^), similar to that of the London urban background site (North Kensington, 10.5 µg m^−3^) and lower than the roadside site (Marylebone Road 21.3 µg m^−3^). BC_880_ concentrations were similar with 0.75 µg m^−3^ to that measured at the North Kensington urban background site (0.7 µg m^−3^), higher than that measured at the rural site Chilbolton (0.29 µg m^−3^) and lower than that measured at Marylebone Road (roadside site, 3.19 µg m^−3^).

#### 3.1.2 Poles Lane campaign

During the sampling campaign in Poles Lane the mean (median) PNC concentration was 7532 (4285) p cm^−3^ (Table 2). These concentrations were lower than those found during the campaign in Horley but were comparable to the concentration found at Oaks Road near Heathrow in airport in 2016, with mean (median) concentration of 7408 (3948) (Stacey et al. 2020). The concentrations during the campaign at Poles Lane were lower than those found in Harlington near Heathrow airport, during both the warm (19,000 p cm^−3^) and cold season (22,000 p cm^−3^) by Masiol et al (2017).

Fig. 2 summarises the particle size distribution, as found during the campaign in Poles Lane, the mean and median size distributions were dominated by the nucleation mode (14–30 nm). However, the median size distribution shows no clear mode and is elevated throughout the nucleation and Aitken mode (14–100 nm).

Mean (and median) particle number concentrations were compared to national network SMPS measurements for a short, overlapping period during the campaign in Poles Lane (see Fig. S3). Concentrations measured at Poles Lane during the campaign 11,918 (7383) p cm^−3^ were higher than those measured at the rural site at Chilbolton 3400 (3083) p cm^−3^ and the London urban background site at North Kensington 6081 (4686) p cm^−3^. The mean concentrations were similar to the mean concentrations measured (11,641 p cm^−3^) at Marylebone Road in London, whereas the median concentrations were lower than those measured at Marylebone Road (10,600 p cm^−3^). This difference is caused by much higher maximum concentration measured during the campaign at Poles Lane (76,066 p cm^−3^) compared to Marylebone Road (34,832 p cm^−3^) but generally lower concentration for most of the period (Fig. S3) as the site is upwind of the airport and major roads the majority of the time.

As found during the campaign in Horley, diurnal profiles of the particle number concentrations (Fig. S4) showed elevated levels during rush hours in the morning and evening. The concentrations stayed elevated during the day but not as noticeably as during the campaign in Horley. The concentrations fell to their minimum during the early morning hours, reflecting reduced airport activity and associated traffic.

The mean and median energy averaged noise levels (L_Aeq-1hr_) were 58 dB, which was higher than those measured at Horley and reflect the closer proximity to the airport perimeter and runway. The diurnal profiles of the noise reflect the diurnal profiles of the flight numbers (Figs. S5 and S6). The median flight numbers during the Poles Lane campaign were 37 flights per hour (diurnal variation of flights is given in Fig. S6); the maximum flight numbers per hour were 56, which was the same as during the campaign in Horley.

The mean NO_2_ concentration measured during the campaign at Poles Lane was 19.1 µg m^−3^. This was higher than the concentrations found at the rural site (Chilbolton 10.3 µg m^−3^) but lower than that found at the urban background site North Kensington (35.7 µg m^−3^) or the roadside site London Marylebone Road (71.4 µg m^−3^). There were no measurements of PM_10_ at Poles Lane. The mean BC_880_ concentration during the campaign was 0.64 µg m^−3^, which was higher than that measured at the rural site (0.51 µg m^−3^) but lower than the concentrations measured at the urban background site (1.13 µg m^−3^) or the roadside site (2.75 µg m^−3^).

### 3.2 Source factors and contributions

**Fig. 3:**
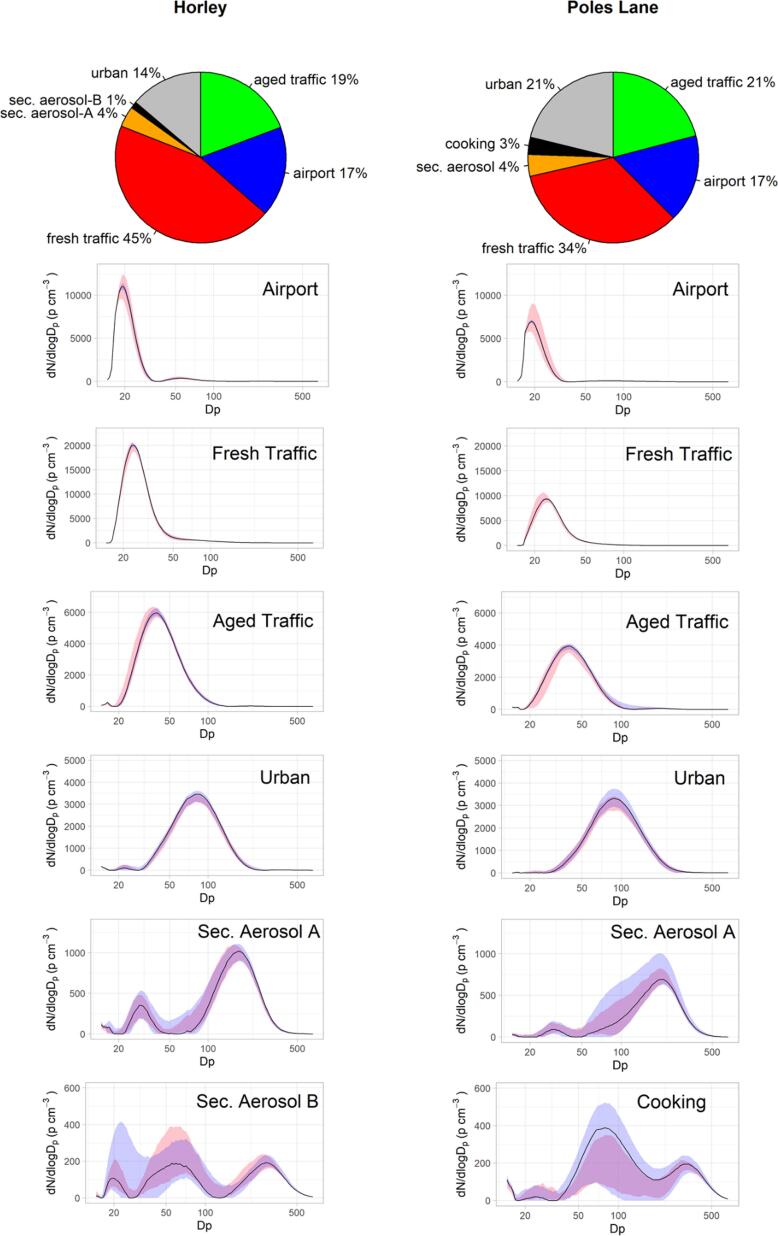
Factor contribution to particle number concentration in percentage and factor profiles with error estimate (bootstrap 25^th^–75th percentile = pink; displacement error min and max = purple) for Horley (left) and Poles Lane (right). (For interpretation of the references to colour in this figure legend, the reader is referred to the web version of this article.)

**Fig. 4:**
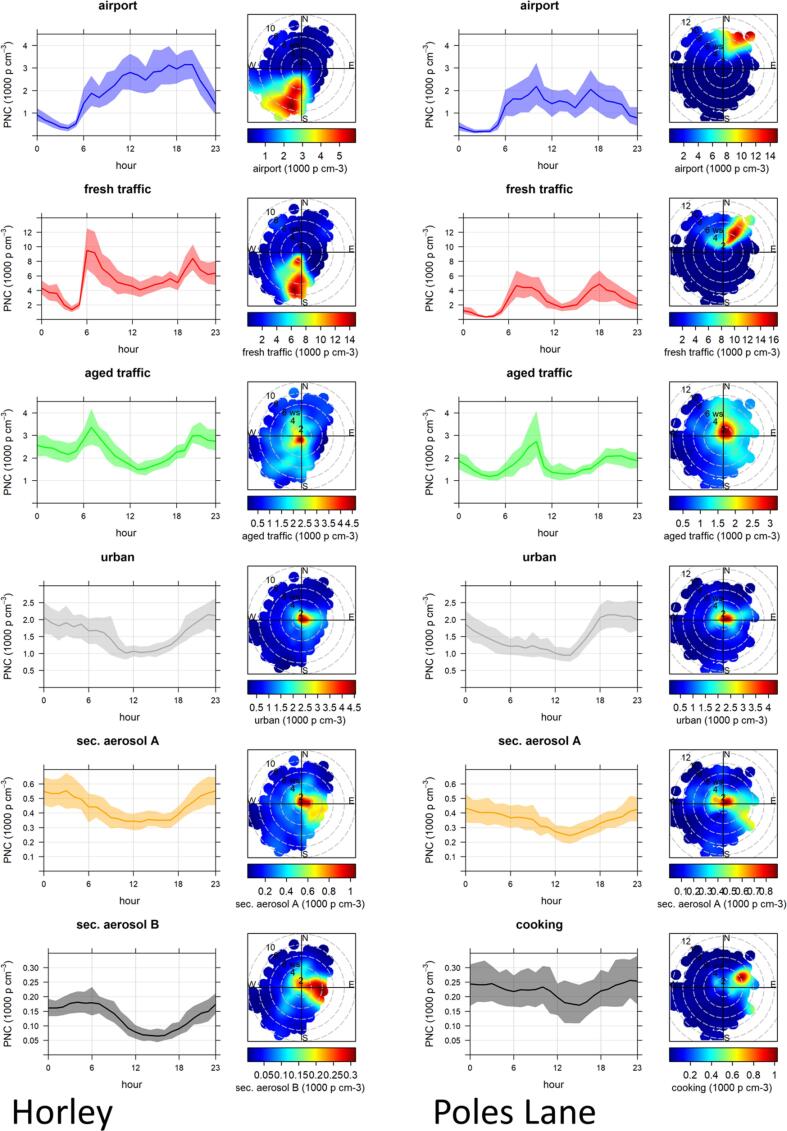
Diurnal variation and polar plots of identified factors for Horley (left) and Poles Lane (right).

A six-factor solution was chosen as the optimum solution for both sites; this solution had the most physically meaningful source profiles and temporal behaviour when compared to solutions with higher or lower number of factors. Further information on the determination of the solutions can be found in supplement S2.2. Table 3 summarises the factor contributions, displacement (DISP.) ranges, bootstrap results (25th and 75th percentile) and the number of modes and mode peak size for each factor. Factor contribution to PNC in percentage and factor profiles with error estimate are shown in Fig. 3, while diurnal variations and polar plots of identified factors are shown in Fig. 4. All factors are discussed in detail below.

**Table 3:** Summary of PMF results for both sites: Percentage factor contribution (base case, Disp. range and bootstrap (25th-75th percentile) results, number and peak of modes.

		**% Contribution**					Number of Modes*	Peak of Mode(s)** (nm)
		**base case**	**DISP. range**		**bootstrap**			
					**(25th percentile)**	**(75th percentile)**		
**Horley**	**F1: airport**	**17.1**	16.8	17.2	16.2	18.0	3	**19.5**, 53.3, 241.4
	**F2: fresh traffic**	**44.6**	42.3	46.4	45.6	42.3	1	**24.1**
	**F3: aged traffic**	**19.3**	19.1	19.5	20.1	20.0	2	16.3, **40**
	**F4: urban**	**13.8**	14.0	12.9	13.4	12.9	2	14.6, **82**
	**F5: sec. aerosol A**	**3.9**	5.3	3.2	3.6	4.7	3	14.6, **30**, **174.7**
	**F6: sec. aerosol B**	**1.3**	2.6	0.7	1.0	2.1	4	14.6, **19.5**, **55.2**, **289**

**Poles Lane**	**F1: airport**	**16.6**	15.4	17.3	16.0	19.4	2	**18.8**, 85.1
	**F2: fresh traffic**	**33.9**	30.9	36.6	36.6	33.2	1	**25.0**
	**F3: aged traffic**	**20.9**	20.7	21.7	20.1	19.5	3	14.6, **40**, 201.7
	**F4: urban**	**21.2**	21.7	20.1	21.8	19.7	2	16.3, **88.2**
	**F5: sec. aerosol A**	**4.2**	7.2	3.4	4.1	5.4	3	14.6, 30, **209.1**
	**F6: cooking**	**3.3**	4.1	1.0	1.4	2.8	3	14.6, **79.1**, **333.8**

* only modes above disp. range are listed; **main modes in bold/underlined

#### 3.2.1 Factor 1: Airport

The factor associated with the airport during both campaigns had a mode of particle diameter 19.5 and 18.8 nm at Horley and Poles Lane, respectively (Table 3, Fig. 3), and most likely includes emissions from aircraft as well as other airport activity. A mode below 20 nm has been associated with airport emissions in previous aircraft emission studies and also in source apportionment studies in urban areas near airports (Stacey, 2019, Masiol et al., 2017, Keuken et al., 2015, Rivas et al., 2020). This factor had a mean concentration of 2000 p cm^−3^ during the campaign at Horley and 1200 p cm^−3^ during the campaign at Poles Lane and contributed 17% at both sites (Fig. 3, Table 3). This contribution is lower than reported in previous studies: Masiol et al. (2017) found the contribution of an airport associated factor to be 32–33% near Heathrow airport, measured in two seasons. In a previous source apportionment study near Venice airport (Masiol et al. 2016) the factor associated with the airport contributed 20% to the total PNC.

As shown in supplement (Supplement Table S2), at Horley this factor was moderately correlated with the factor that was identified as fresh traffic, whereas at Poles Lane this factor had a strong correlation with the fresh traffic factor and a moderate correlation with NO_2_ (Supplement Table S2). The diurnal profile shows that the concentrations of this factor started to rise at around 06:00 at both sites and started to drop in the evening when flight numbers decreased (Fig. 4). The lowest concentrations were seen at around 04:00 GMT at Horley site and between 01:00 to 04:00 GMT for Poles Lane. The concentrations of this factor approximately follow the flight activity at Gatwick (Fig. S6); during the campaign at Poles Lane the flight schedule had changed, and thus fewer flights were seen during the night. The polar plots (Fig. 4) show that for both sites this factor had the highest concentrations when the wind was coming from the direction of the airport. Horley site is often downwind of the airport as the predominant wind direction is from the south-west, whereas Poles Lane is predominantly upwind form the airport. These insights support the interpretation of this factor as airport related.

Due to its size range, this factor could be influenced by nucleation events though no substantial events were seen. Masiol et al. (2017) analysed a large regional nucleation event during the sample campaign near Heathrow and concluded that nucleation events may influence PMF results and may “lead to an ‘additive’ bias” but that local sources will overwhelm these regional events. In a multicity study, Rivas et al. (2020) found evidence that airport emissions influenced concertation of nucleation mode aerosols at urban background stations located few kms away.

#### 3.2.2 Factor 2: Fresh traffic

Two factor profiles were identified as traffic related and were very similar at both sites: Fresh Traffic and Aged Traffic (see 3.2.3). Fresh Traffic has one size mode; this was of 24.1 nm and 25 nm at Horley and Poles Lane, respectively. This was lower than in a study near Venice airport, where the factor associated with traffic had a size range between 35 and 40 nm (Masiol et al. 2016), but compared well with a source apportionment study near Heathrow airport where a factor associated with fresh traffic was found in the size range 18–35 nm and 20–35 nm in the cold and warm season, respectively (Masiol et al. 2017). A study in Rochester, NY found a factor in the size range of 20–30 nm, which they associated with traffic (Kasumba et al. 2009), and a study looking at SMPS data from a number of cities across Europe found fresh traffic was associated with size ranges between 13 and 37 nm (Rivas et al 2020).

This factor correlated moderately with NO_2_ at both sites, which is a primary road traffic tracer but showed no or only weak correlations with other primary road traffic tracers, such as NO_X_ and BC_880_ (Supplement Table S2). This has been observed in other studies ((Masiol et al., 2016, Harrison et al., 2011, Vu et al., 2016), where these correlations were weak even though other information is consistent with traffic sources.

Fresh Traffic had the highest percentage contribution during both campaigns. With a mean concentration of 5300 p cm^−3^ it contributed 45% to the total number concentration at Horley. At Poles Lane this factor contributed 34% and had a concentration of 2600 p cm^−3^. This was consistent with previous studies where source associated with traffic emissions also showed high contributions to the particle number concentration: e.g. 28–35% near Heathrow airport (Masiol et al. 2017), 31% and 41% annually for a London background and London roadside site respectively (Rivas et al. 2020). Ogulei et al. (2007) found this traffic factor to contributed between 22 and 31% in different seasons in Rochester, NY.

The polar plots in Fig. 4 show that during the campaign in Horley the highest concentrations for this factor were seen when the wind direction was south/south west, bringing air from the A23 and M23 feeder road, which are the main access roads towards the airport. Further, there was a local contribution from the road adjacent to the monitoring location. During the campaign at Poles Lane the main contribution to the Fresh Traffic factor was during low wind speeds (∼4 m^−s^) from the north easterly wind direction; consistent with an airport car park and a small business area. The diurnal variation of the Fresh Traffic factors showed clear rush hour peaks at both sites. During the Horley campaign, a sharp rise in concentration was observed in the morning when the rush hour traffic and airport traffic coincided, the concentrations then stayed slightly elevated throughout the day and a smaller rush hour peak was seen in the evening. The concentrations reached a minimum at 03:00 to 04:00 GMT when there were few or no flights, thus highlighting the contribution the airport activity had to local traffic. During the Poles Lane campaign both rush hour peaks were pronounced with a drop in concentration during midday and a further drop during the early morning hours.

#### 3.2.3 Factor 3: Aged traffic

A factor which was interpreted as Aged Traffic was also present at both sites with a broad size distribution peaking at 40 nm. Aged traffic particles contributed 19% and 21% at during the Horley and Poles Lane campaigns, respectively, with a mean concentration of 2300 p cm^−3^ and 1700 p cm^−3^. This factor was moderately correlated to NO, NO_2_, NO_X_ and BC_880_ during the campaign in Horley. During the campaign in Poles Lane, this factor was moderately correlated with the factor that was identified as general urban particles and it was moderately to strongly correlated with combustion markers (NO, NO_2_, NO_X_ and BC_880_, BC_370_). (Vu et al. 2016) also identified a second/aged traffic factor in a study in London in the size range between 20 nm and 100 nm (peak number diameter at 52 nm), which correlated to other combustion markers; it contributed around 30% to the total UFP. During the Heathrow study by Masiol et al (2017) a factor in the size range of 28–60 nm and a contribution of around 19% was similarly identified as an aged traffic signal. Ogulei et al. (2007) identified a second traffic factor in the size range of 50–80 nm in a study in Rochester, NY.

Polar plots reveal that this factor contributed most during calm conditions, with contributions from the general urban area and road networks at higher wind speeds. Diurnal plots show two rush hour peaks, with the peaks appearing later than those for the fresh traffic factor, underpinning the interpretation that this was an aged signal. This was especially noticeable for the morning peak during the Poles Lane campaign, which was undertaken during colder weather. The concentrations for this factor remained elevated throughout the night at both sites.

#### 3.2.4 Factor 4: Urban background

The fourth common factor identified during both campaigns was attributed to a general urban background source. This factor had dominant modes of 82 nm and 88.1 nm at Horley and Poles Lane, respectively (Fig. 3). The mean concentrations were 1600 and 1500 p cm^−3^, which represents 14% and 21% of the particle number concentration for Horley and Poles Lane, respectively (Table 3). This factor was strongly to very strongly correlated with the black carbon concentration and a secondary aerosol factor identified at both sites. It was further moderately to strongly correlated with general combustion markers (NO, NO_2_, NO_X_) during the Poles Lane campaign and moderately correlated with NO and NO_X_ during the Horley campaign (Supplement Table S2). The concentrations of this factor were elevated in calm conditions and therefore likely local in origin. The diurnal pattern of these factors was only slightly influenced by the rush hour; the main influence on the diurnal profile were mixing layer dynamics with the lowest concentrations around midday and elevated concentrations during the night. At Poles Lane these concentrations rose quickly in the early evening, which might reflect a contribution of emissions from domestic space heating as this campaign took place during a colder period.

Masiol et al (2017) found a similar factor in the size range of around 80 nm (50–150 nm in the warm season and 55–170 nm in the cold season) which contributed 14% to their overall UFP in the warm season and 8% in the cold season. They identified this factor as urban accumulation, and it was linked to combustion sources. Another similarity was that the concentrations were higher during the night than daytime. Rivas et al. (2020) also identified a factor in London with a broad main mode of 80.6 nm, which was dominated by traffic emissions with influences from biomass burning and other urban sources. Especially in winter, this factor had a prolonged evening peak at the London background site, which is similar to the prolonged peak seen at Poles Lane. Kasumba et al. (2009) identified a bimodal factor in Rochester, NY, with the main mode at around 70–90 nm, which was interpreted as a second traffic factor as it has a clearer source directionality as the factor identified in this study.

#### 3.2.5 Factor 5: Secondary aerosol

A multimodal factor considered to be secondary aerosol has been identified for both sites. The main mode was found at 174.7 nm for Horley and 209.1 nm at Poles Lane, with a second mode in the Aitken range and a small third mode in the nucleation range at both sites. This factor represented only a small fraction of the particle number concentration for both sites (Horley: 4%; Poles Lane 4%) and had a mean concentration of 440 and 350 p cm^−3^ at Horley and Poles Lane, respectively. This factor had a strong to very strong correlation with black carbon and the urban factor at both sites (Supplement Table S2). It was further moderately correlated with a second secondary aerosol factor during the Horley campaign and moderately correlated to NO, NO_X_ and the volatile PM_10_ fraction during the Poles Lane campaign. The wind directionality showed increased concentrations during calm conditions, which might indicate secondary aerosols formed from local sources, and at slightly higher wind speeds during easterly winds, with air masses travelling from other areas of the UK and continental Europe. The diurnal pattern of this factor was mainly influenced by mixing layer dynamics at both sites with the lowest concentrations during midday and the afternoon.

Secondary aerosol factors were also identified in two Rochester, NY studies (Ogulei et al., 2007, Kasumba et al., 2009). However, they identified different secondary aerosol factors (sec. nitrate, sec. sulphate and O_3_ – rich sec. aerosol) – these factors were all multimodal with main modes in the higher size fraction (100–300 nm). Ogulei et al. (2007) suggested that the multimodal structure of this secondary factor might indicate both local and distant sources might contribute to particles in this factor. Rivas et al. (2020) found one secondary aerosol factor in the size range of 93–294 nm in different European cities; in London this factor was found to be lowest in the afternoon, due to thermally instable components, such as ammonium nitrate and semi volatile organic aerosols and mixing layer height.

#### 3.2.6 Factor 6: Site specific factors

A sixth factor, present at both sites, was also multimodal with a mode in the higher size fraction (Horley: peak at 289 nm, Poles Lane: peak at 333.8 nm) having the least rotational ambiguity, indicated by the smaller error estimates in Fig. 3. Further modes were found in the Aitken and nucleation modes, but these had higher uncertainties Table 3. Masiol et al. (2017) also found two secondary aerosol factors, which were multimodal with smaller modes showing higher rotational ambiguity, indicated by higher DISP (displacement) ranges.

This factor, however, had different characteristics at the two sites. During the Horley campaign this factor had a very small contribution to the total particle number concentration (1%) and was low in concentration (130 p cm^−3^). This factor was moderately correlated with BC_880_ and BC_370_ and volatile PM_10_, as well as the secondary aerosol factor. Meteorological data showed that the concentration was highest with easterly wind directions. The diurnal profile was predominantly influenced by mixing layer dynamics and thus this factor has also been interpreted as an additional secondary aerosol factor. The highest concentrations coincided with higher wind speeds than for the first secondary aerosol factor and thus this might indicate that the origins were less local. This interpretation was supported by the higher correlation with volatile PM fraction.

At Poles Lane this factor was also multimodal. It was the factor with the smallest contribution to the particle number concentration (3%; concentration: 220 p cm^−3^) at this site. This factor was moderately correlated to NO_2_ and NO_X_ and strongly correlated with BC_880_ and BC_370_ and volatile PM_10_. The diurnal profile was slightly influenced by mixing layer dynamics but the variation during the day was not very pronounced. Looking at the polar plots there appeared to be a distinct source to the north-east of the site, which caused the concentration to be highest when the wind speed was around 4 ms^−1^. This put the source slightly east of the fresh traffic source, which also peaked at around 4 ms^−1^. Due to the directionality, this factor was thought to be associated with cooking aerosol as there is a facility preparing airline food next to the car park, which is responsible for the on-flight catering for Gatwick airport flights (personal communication).

In a study undertaken using ambient data at a busy road in London, (Harrison et al. 2011) found a factor with a mode slightly larger than 20 nm, which due to diurnal variation and source direction was interpreted as cooking aerosol. In a study looking at the particle size distribution in an occupied townhouse, (Ogulei et al. 2006) found that frying tortillas resulted in a particle number peak at around 90 nm, where broiling fish resulted in a lower mode (50 nm). The main and least rotationally ambiguous mode was much higher (289 nm, 333.8 nm) during this study but reviews on cooking aerosol by (Abdullahi et al. 2013) and on particle number concentrations by (Vu et al. 2015) indicate that cooking aerosols are associated with a multitude of size fractions, depending on the cooking process.

### 3.3 Aircraft factor and runway direction

Depending on wind direction Gatwick airport either operates in westerly or easterly mode. In westerly mode, during westerly wind direction, planes take off toward the west (into the wind) and arrive from the east. In easterly mode, during easterly wind directions, planes take off towards the east and land from the west.

**Fig. 5:**
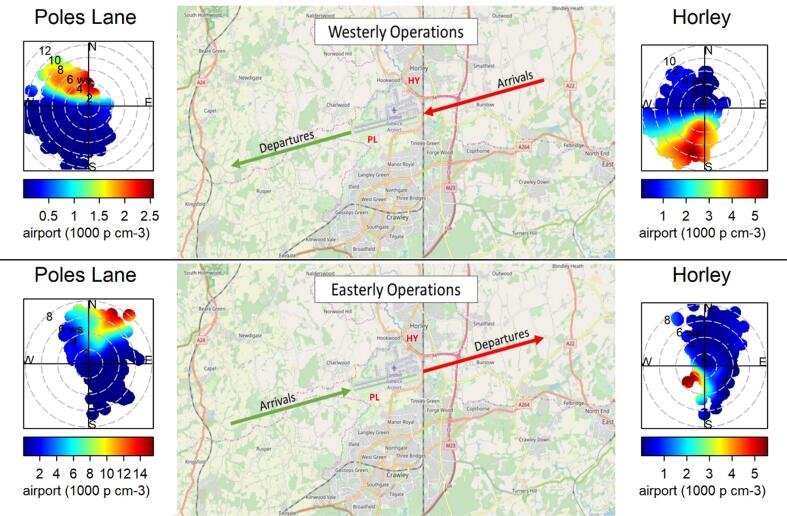
Airport operation (Gatwick Airport Flight Performance Team 2018) and polar plots of airport factor during easterly and westerly operations in each campaign (Horley – right hand graphs; Poles Lane – left hand graphs).

During the Horley campaign 79% of the time the airport operated in westerly direction, 19% in easterly direction and only in 2% of the time there were no flights. Fig. 5 shows polar plots of the airport factor separated by operation mode and it is clear that during the measurement campaign in Horley westerly operation had the biggest influence on the site and caused the highest airport contributions to PNC recorded in terms of mean and median (Table 4) as it coincided with the main wind direction. The lowest airport contribution was recorded when no flights were taking off/landing. It was based on very few measurements but might indicate that other services operating at that airport contributed to this factor.

**Table 4:** Airport factor contribution split by airport operation.

	**Hourly Concentration at Horley Site (p cm^−3^)**		
	**westerly operations**	**easterly operations**	**no flights**
1st Qu.	220	80	−5
**Median**	**1100**	**190**	**48**
**Mean**	**2300**	**550**	**120**
3rd Qu.	3100	470	170
N	1612	398	22

	**Hourly Concentration at Poles Lane Site (p cm^−3^)**		
	**westerly operations**	**easterly operations**	**no flights**

1st Qu.	23	160	−3
**Median**	**89**	**480**	**47**
**Mean**	**560**	**2600**	**100**
3rd Qu.	310	2700	140
N	1310	762	165

**Table 5:** Pearson’s R and Spearman’s Rho pairwise correlations between L_Aeq-1hr_ noise levels and measurements of airport activity, meteorology, or source specific particle number concentrations, where P ≤ 0.05.

**Parameter**		**Horley**			**Poles Lane**		
**Group**	**Type**	**Count**	**R**	**Rho**	**Count**	**R**	**Rho**
Airport activity	Total flights (n)	2,058	0.51	0.49	1,964	0.52	0.52
	Easterly flights (n)	367	0.44	0.42	609	0.55	0.49
	Westerly flights (n)	1,368	0.34	0.27	1,037	0.32	0.29
Meteorology	Air temperature (°C)	2,026	0.30	0.38	2,022	0.29	0.28
	Precipitation (mm)	1,956	0.17	0.06	1,495	0.12	0.21
	Relative humidity (%)	2,026	−0.27	−0.33	2,022	−0.25	−0.27
	Wind speed (m s^−1^)	2,026	0.62	0.56	2,022	0.65	0.55
Primary PNC sources	Total PNC (p cm^−3^)	2,033	0.30	0.33	2,022	0.10	–
	Airport PNC (p cm^−3^)	2,033	0.42	0.52	2,023	0.18	0.17
	Fresh traffic PNC (p cm^−3^)	2,033	0.30	0.38	2,023	0.16	0.14
	Urban PNC (p cm^−3^)	2,033	−0.17	−0.24	2,023	−0.22	−0.31

**Table 6:** OLS multivariate regression models of L_Aeq-1hr_ noise measurements, meteorology and particle number concentrations.

**Parameter (Normalised 0**–**1)**		**Horley: Coefficients**			**Poles Lane: Coefficients**		
**Group**	**Type**	**Model 1**	**Model 2**	**Model 3**	**Model 4**	**Model 5**	**Model 6**
Background L_Aeq-1hr_ (dB)	Intercept	50.71	55.58	46.87	52.04	59.77	46.46
Meteorology	Air temperature (°C)	2.72	–	2.84	(n/s)	–	4.11
	Precipitation (mm)	2.66	–	1.44	(n/s)	–	(n/s)
	Relative humidity (%)	(†)	–	–	(n/s)	–	1.78
	Wind speed (m s^−1^)	15.61	–	18.54	20.26	–	23.55
	Wind direction: NE vs. SW	−2.29	–	−1.1	−0.64	–	−2.19
	Wind direction: NW vs. SW	−3.89	–	−2.59	−0.75	–	−1.17
	Wind direction: SE vs. SW	1.79	–	1.89	−2.6	–	−2.93
Primary PNC sources	Airport PNC (p cm^−3^)	–	13.01	4.02	–	3.38	7.33
	Fresh traffic PNC (p cm^−3^)	–	4.26	10.35	–	4.16	(*)
	Urban PNC (p cm^−3^)	–	−5.47	8.98	–	−10.49	12.24
Regression Statistics	Adjusted R-squared	0.56	0.2	0.65	0.49	0.08	0.55
	F-Test (p-value)	< 0.01	< 0.01	< 0.01	< 0.01	< 0.01	< 0.01
	Kolmogorov-Smirnov (p-value)	0.52	< 0.01	0.33	0.8	< 0.01	0.61

(n/s) Non-significant, where P > 0.05

(†) Variable omitted due to high correlation with air temperature (R > 0.6)

(*) Variable omitted due to high correlation with airport particulates (R > 0.6)

During the campaign in Poles Lane the airport operated in westerly operation 59% of the time, in easterly operation for 34% of the time and in 7% of the time there were no flights. This campaign was carried out during the winter schedule of the airport, during which fewer flights were recorded at night-time. The highest mean and median concentrations were recorded during easterly operation and the lowest concentrations were recorded with no flight movement present (Table 4).

Stacey (2019) found that departing aircrafts emitted higher numbers of particles than arriving aircrafts at Heathrow airport, which has separate runways for arrivals and departures. A study in Los Angeles (Hsu et al. 2013), found that take-off activities result in a large contribution to UFP counts near runway but that these contributions decrease with distance. They also noted that as take-offs occur facing the wind, communities downwind of the airport will be subject to high UFP concentrations. At Gatwick, aircraft were departing and arriving on a single runway; a second runway immediately next to the primary runway is only used if the primary runway is out of action. Since take-off and landing were always occurring on the same runway, we were not able to distinguish between their respective emissions. At Boston’s Logan airport, Hudda et al. (2020) observed that UFP concentrations were greater during overhead landing operations compared with take offs. They suggested that this may be due to shallow angles of approach, compared with take-off and vortices from descending aircraft wingtips, which may bring emissions to ground. This may explain the measurement of high concentrations of airport UFP at Horley from the south and south-southeast (Fig. 5).

### 3.4 Aircraft factor and noise

Hourly L_Aeq-1hr_ noise measurements were found to have a moderate linear correlation (0.4–0.6) with the total number of flights at both Horley and Poles Lane (Table 5). Differentiating between the directions of operational activity, it was observed that easterly operations had a higher correlation with noise than their westerly counterparts, this was reflected in bivariate polar plots given in Supplement Fig. S8.

Noise transmission through the air is influenced by meteorological parameters, which was demonstrated by the correlations found: there was a strong linear correlation (0.6–0.8) between L_Aeq-1hr_ noise and wind-speed; there was a weak linear and non-linear correlation (0.2–0.4) between L_Aeq-1hr_ noise and the meteorological influences of air temperature and relative humidity; precipitation had a weak linear correlation with noise at Horley, and a very weak non-linear correlation (<0.2) with noise at Poles Lane.

Airport particle counts and measured noise levels appear to follow a non-linear relationship, with moderate correlations reported at Horley (0.4 – 0.6) and weak correlations at Poles Lane (<0.2). Road-transport particulate counts are weakly correlated with noise at both sites (0.2–0.4).

Ordinary least squares (OLS) regression models were used to control for these complex influences on noise, and to understand the interaction between noise and source dependent particle number counts (Table 6). The predictor variables have been normalised on a 0–1 scale, to adjust for any disparities in variable size, ensuring that the regression model coefficients (effect sizes) are in proportion with one another: the size of the coefficient directly indicates the importance of a parameter.

Models 1 (Horley) and 4 (Poles Lane) examine the collective influence of meteorological parameters on L_Aeq-1hr_ noise levels. Meteorological parameters appear to explain 49–56% of the recorded variation in hourly noise. Noise events are primarily associated with increases in wind speed and changes in wind direction at both sites. Noise events are also influenced by temperature and rainfall at Horley, but not at Poles Lane.

Models 2 (Horley) and 5 (Poles Lane) examine how L_Aeq-1hr_ noise levels may relate to nearby sources of air pollution, which originate from aircraft, road-transport and other urban activities. These anthropogenic activities appear to explain 20% of the variation in hourly noise at Horley, and 8% of the variation at Poles Lane. However, the Lilliefors Kolmogorov-Smirnov tests indicate that the residuals in these models are not normally distributed (P > 0.05).

Models 3 (Horley) and 6 (Poles Lane) examine the influence of primary pollution sources on L_Aeq-1hr_ noise levels, adjusting for meteorological influences. Both sites typically record background L_Aeq-1hr_ noise levels of 46 dB. The models appear to explain 55–65% of the recorded variation in hourly noise.

In terms of noise sources, particle number concentrations from fresh traffic were associated with the largest potential increase to background L_Aeq-1hr_ noise levels at Horley (up to 10.35 dB), followed by particle number concentrations from background urban activities (up to 8.98 dB). Particle number concentrations linked to the airport were associated with an increase of noise above background levels of up to 4.02 dB (median = 2.01 dB). At Poles Lane particle number concentrations linked to general urban sources were associated with the highest increase in noise above background level (12.24 dB). Particle number concentrations linked to fresh traffic and airport activity could not be separated for this model due to the high levels of correlation at this site (R > 0.6). Together these particle number sources were associated with a 7.33 dB increase on background L_Aeq-1hr_ noise levels.

Meteorology is shown to substantially modify the influence of these noise sources. The largest increases in detected noise were associated with increases in wind speed, which can cause (a) sound to travel faster and over greater distances, (b) instrument interference, and (c) an increase in background noise levels – e.g. rustling leaves. High wind speeds of 10 m s^−1^can increase L_Aeq-1hr_ noise exposure levels at Horley by 18.54 dB, and at Poles Lane by 23.55 dB (versus calm days of 0.2 m s^−1^). Although high wind speeds may elevate levels of noise exposure, such conditions will increase the dispersion of pollutants, which in part explains why differences in exposure exist.

Wind direction may also influence noise levels, with south-easterly and south-westerly wind directions at Horley (downwind of Gatwick airport), respectively recording L_Aeq-1hr_ noise levels up to 2.59 dB and 4.48 dB above exposures under north-easterly or north-westerly winds. The propagation of sound in a gas is known to be temperature dependent, with higher temperatures increasing the speed of sound. The models show that L_Aeq-1hr_ noise levels measured on the hottest hour at Horley (33 °C) are 2.84 dB louder than on the coldest hour (2 °C), and at Poles Lane there is a 4.11 dB difference between temperatures of 19 °C and −5°C.

The regression models further elaborate on the complexity of the source apportionment of noise. Influenced by a number of activities, and the propagation of which is further determined by multiple meteorological influences.

Air and noise pollution are shown to have separate exposure distributions, although overlap exists under favourable atmospheric conditions. In the UK, anticyclone weather systems form under high pressure systems with light easterly winds. In the warm season these stable air masses cause the air to warm and stagnate, resulting in the build-up of pollutants. A stable atmosphere also favours the propagation of noise, and sound is likely to exhibit an omnidirectional spread where wind impedances are minimal. The bivariate polar plots, given in Supplement Fig. S8, find the highest correlations between noise and air pollution at both sites when wind speeds are < 1 m/s. High correlations are also observed at Horley under low easterly and south-easterly winds (3 m s^−1^).

## 4 Conclusions

Particle size distributions and noise levels were measured at two locations near Gatwick airport.

Mean particle number concentrations at our measurement locations, at ca. 0.6 and 0.3 km from the perimeter of Gatwick Airport were similar to concentrations measured just two metres from the highly trafficked road in central London. Our two measurement campaigns were located at different distances from the runway and terminal areas. Horley was 1.6 km from the eastern end of the runway and just under 1.0 km from the terminals, whereas Poles Lane was closer to the runway (0.6 km from centre of runway) and further from the terminal area (>2 km). Given that peak particle number concentrations during the campaign at Poles Lane were greater than that measured during the campaign at Horley, we can therefore conclude that take-off and landing activities on the runway are a greater source of particle number concentrations compared with taxiing activities near the terminals.

A source apportionment analysis using PMF identified six factors during each campaign. An Airport factor, with consistent size distribution contributed 17% to the particle number concentration during both campaigns. This was most likely due to aircraft engine emissions. But this was not the only source linked to airport activities. At Poles Lane cooking aerosol was detected from airport caterers. Particles linked to fresh and aged traffic emissions dominated the UFP concentration at both sites. At Poles Lane the fresh traffic emissions were clearly linked to a carpark within the airport perimeter. An urban source was identified at both sites along with a secondary aerosol factor. At Horley a further secondary aerosol factor was identified.

Aircraft generally take off into the wind to maximise lift. This behaviour affected the distribution of UFP around the airport, however, the airport factor was greatest when the sites were downwind of the runway.

At both sites noise levels were above noise level recommendations by the WHO (World Health Organisation) for L_den_ (Day-evening-night-weighted sound pressure level) < 45 dB and L_night_ (Equivalent continuous sound pressure level when the reference time interval is the night < 40 dB) (WHO 2018).

At Horley there was a moderate non-linear correlation (0.4 – 0.6) between airport particle counts and noise. Traffic factors had a weak correlation (0.2–0.4) with noise. There were no clear correlations between UFP factors and noise at Poles Lane.

Regression models of UFP sources and noise suggested that the largest source of noise (L_Aeq-1hr_), above background at Horley was associated with fresh traffic and urban UFP rather than UFP from the airport. At Poles Lane the regression model was not able to separate the increased noise associated with fresh traffic and airport sources of UFP. Instead, the urban UFP was associated with the greatest increase in noise.

This clearly shows the complexity of the source apportionment of noise. This most likely reflects the different methods of propagation for air pollution, in our case UFP, and noise, with air pollution dispersion being critically dependent on wind direction.

Generally, there were moderate to low correlations between UFP and noise at the two monitoring sites investigated, which suggests that UFP is unlikely to be an important confounder in epidemiological studies of aircraft noise and health in communities living near airports. However, we found that correlations between UFP and noise can be affected by meteorological factors, which could be important in particular for studies of short-term associations between aircraft noise and health. A more detailed examination of correlations by both space and time is warranted to explore this in more detail.

## CRediT authorship contribution statement

**Anja H. Tremper:** Investigation, Formal analysis, Methodology, Writing – original draft, Writing – review & editing. **Calvin Jephcote:** Methodology, Writing – original draft, Writing – review & editing. **John Gulliver:** Conceptualization, Funding acquisition, Supervision, Writing – review & editing. **Leon Hibbs:** Investigation, Writing – review & editing. **David C. Green:** Investigation, Formal analysis, Methodology, Writing – review & editing. **Anna Font:** Investigation, Methodology, Writing – review & editing. **Max Priestman:** Investigation, Methodology, Writing – review & editing. **Anna L. Hansell:** Conceptualization, Funding acquisition, Supervision, Writing – original draft, Writing – review & editing. **Gary W. Fuller:** Conceptualization, Funding acquisition, Investigation, Methodology, Supervision, Writing – original draft, Writing – review & editing.

## Declaration of competing interest

The authors declare that they have no known competing financial interests or personal relationships that could have appeared to influence the work reported in this paper.
