# Supplementary material for: Sources of particle number concentration and noise near London Gatwick Airport
Source: Environ Int. 2022 Mar;161:107092. doi: 10.1016/j.envint.2022.107092 (PMC8885425; doi:10.1016/j.envint.2022.107092)
Supplement: Supplementary data 1 [file mmc1.docx]

**Supplement:**

**Sources of particle number concentration and noise near London Gatwick Airport**

Anja H. Tremper^a^*, Calvin Jephcote^b^, John Gulliver^b^, Leon Hibbs^c^, David C. Green^a^, Anna Font^a^, Max Priestman^a^, Anna L. Hansell^b^, Gary W. Fuller^a^

*^a^ MRC Centre for Environment and Health, Environmental Research Group, Imperial College London, London, UK*

*^b^ Centre for Environmental Health and Sustainability, University of Leicester, Leicester, UK*

*^c^ Environmental Health, Reigate & Banstead Borough Council, Town Hall, Reigate, Surrey, UK*

*Corresponding author: Dr. Anja H. Tremper ([anja.tremper@imperial.ac.uk](mailto:anja.tremper@imperial.ac.uk))

**Supplement 1. METHODOLOGY**


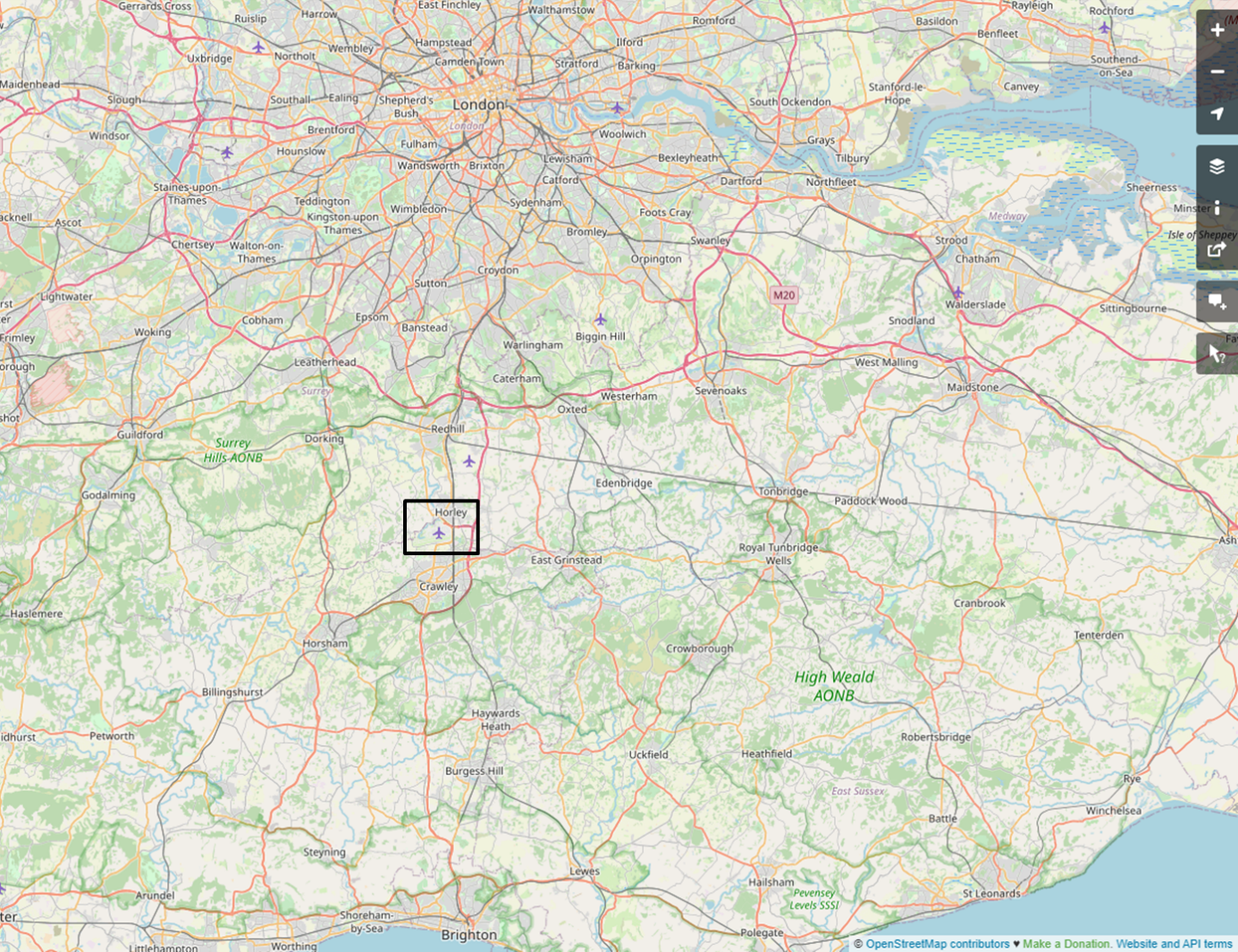


Figure S1: Map of South East England with indication of study area around Gatwick airport

**Supplement 2. RESULTS AND DISCUSSION**

**Supplement 2.1.: Overview**


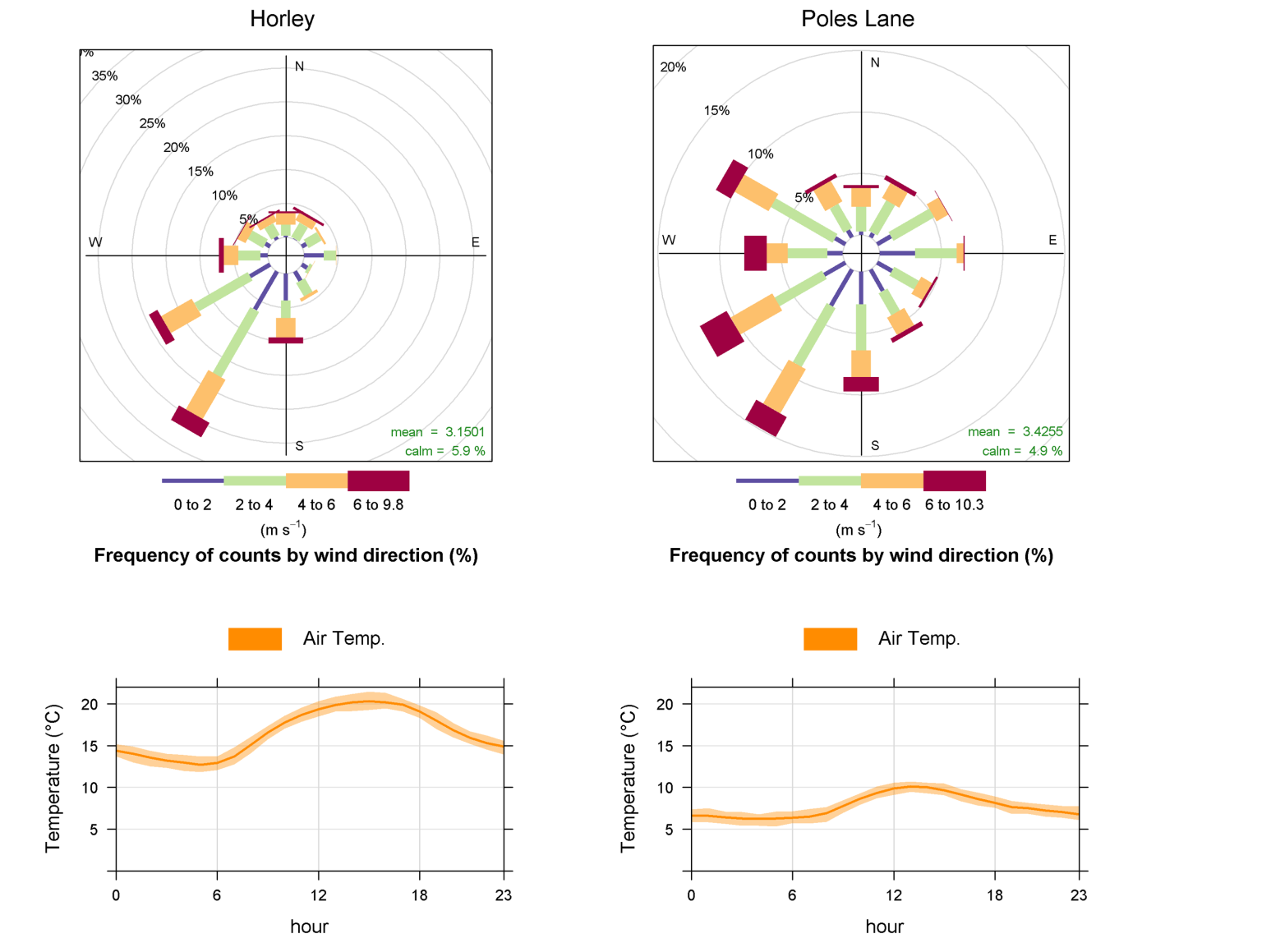


Figure S2: Wind Roses and diurnal air temperature profile during the sampling campaigns in Horley (left) and Poles Lane (right)


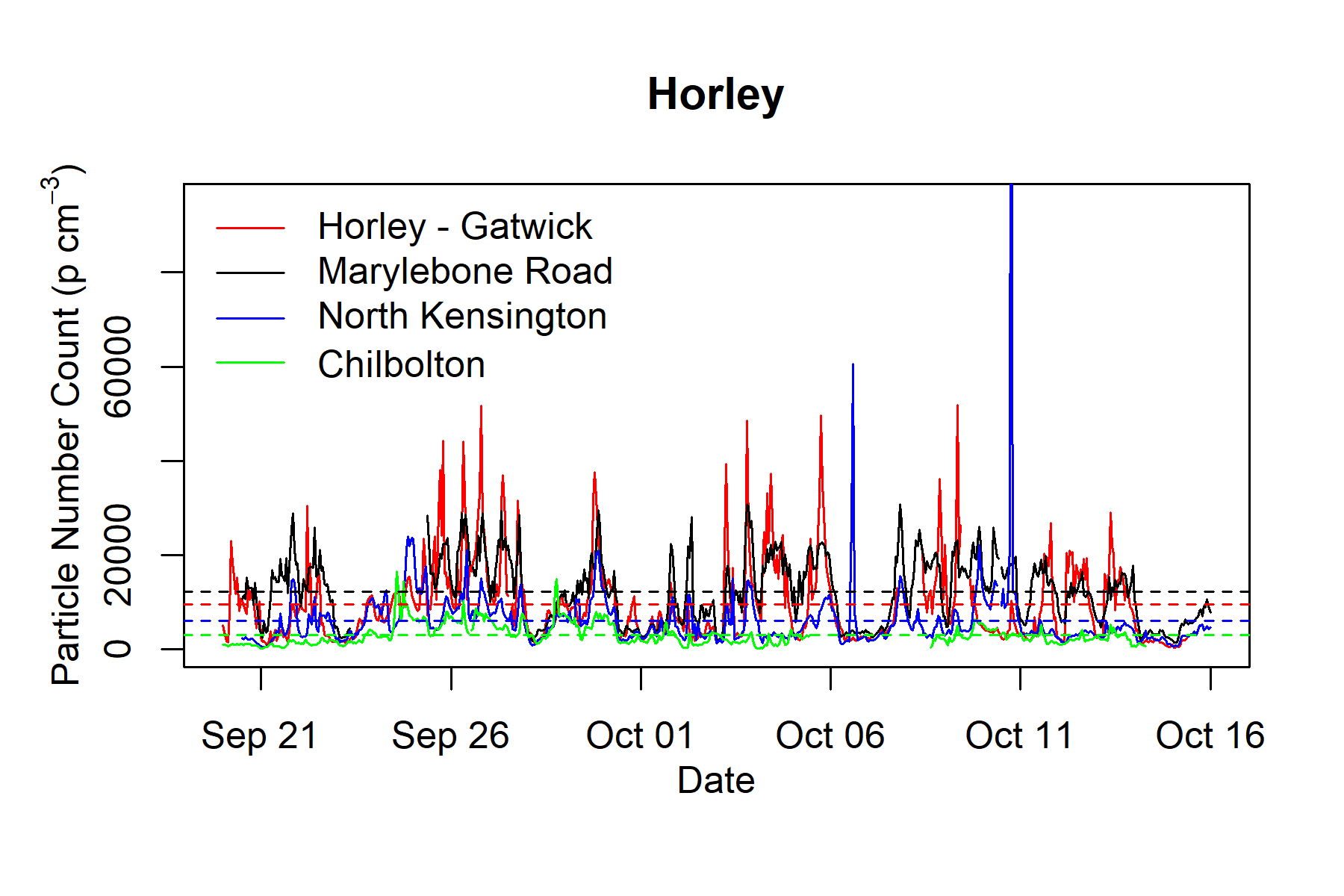

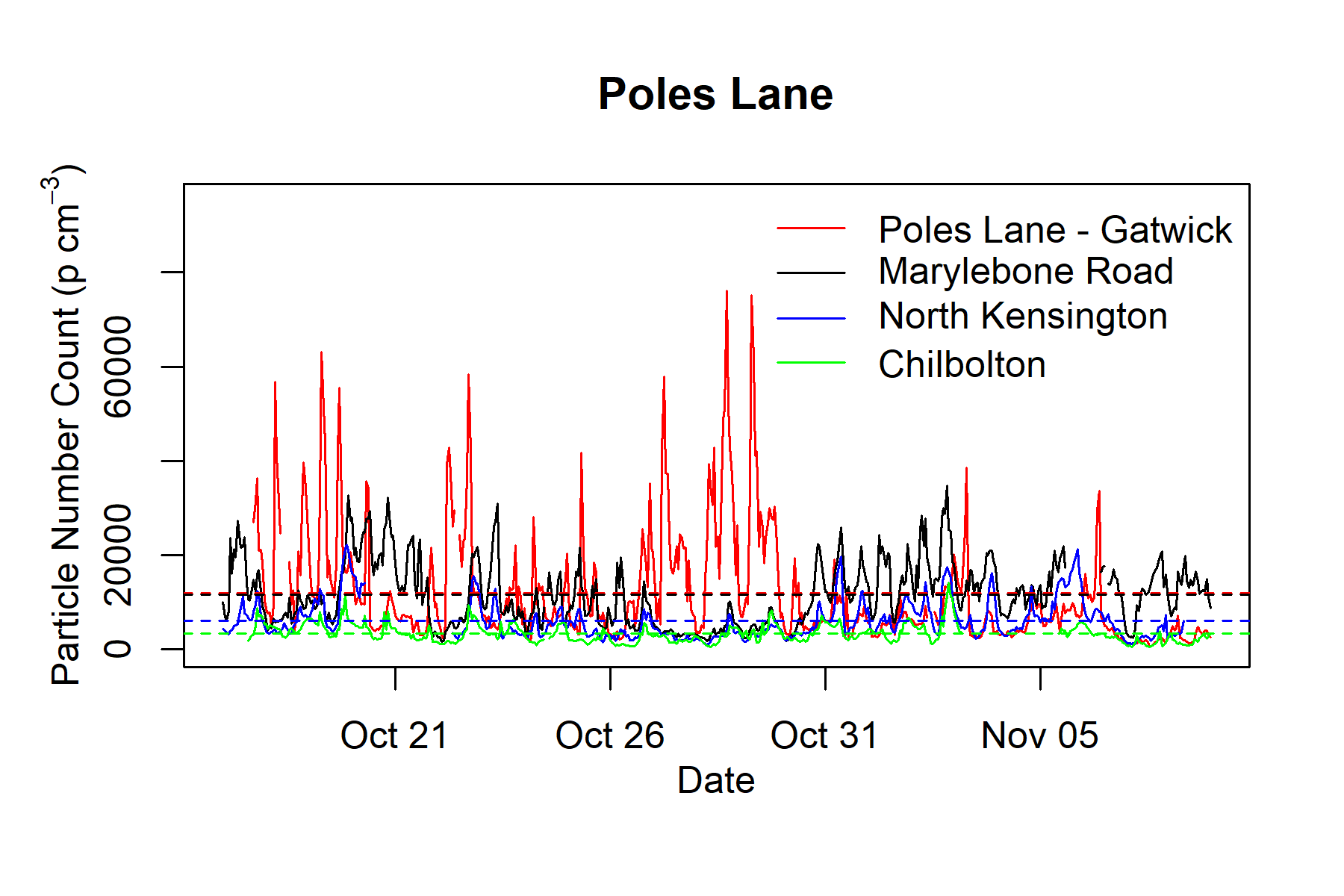


Figure S3: Total UFP concentrations measured by SMPS in Horley (top) and Poles Lane (bottom) in comparison to measurements at a roadside site (Marylebone Road) and background site (North Kensington) in London and a rural site in Chilbolton; dashed lines represent the mean concentration (p cm^-3^).


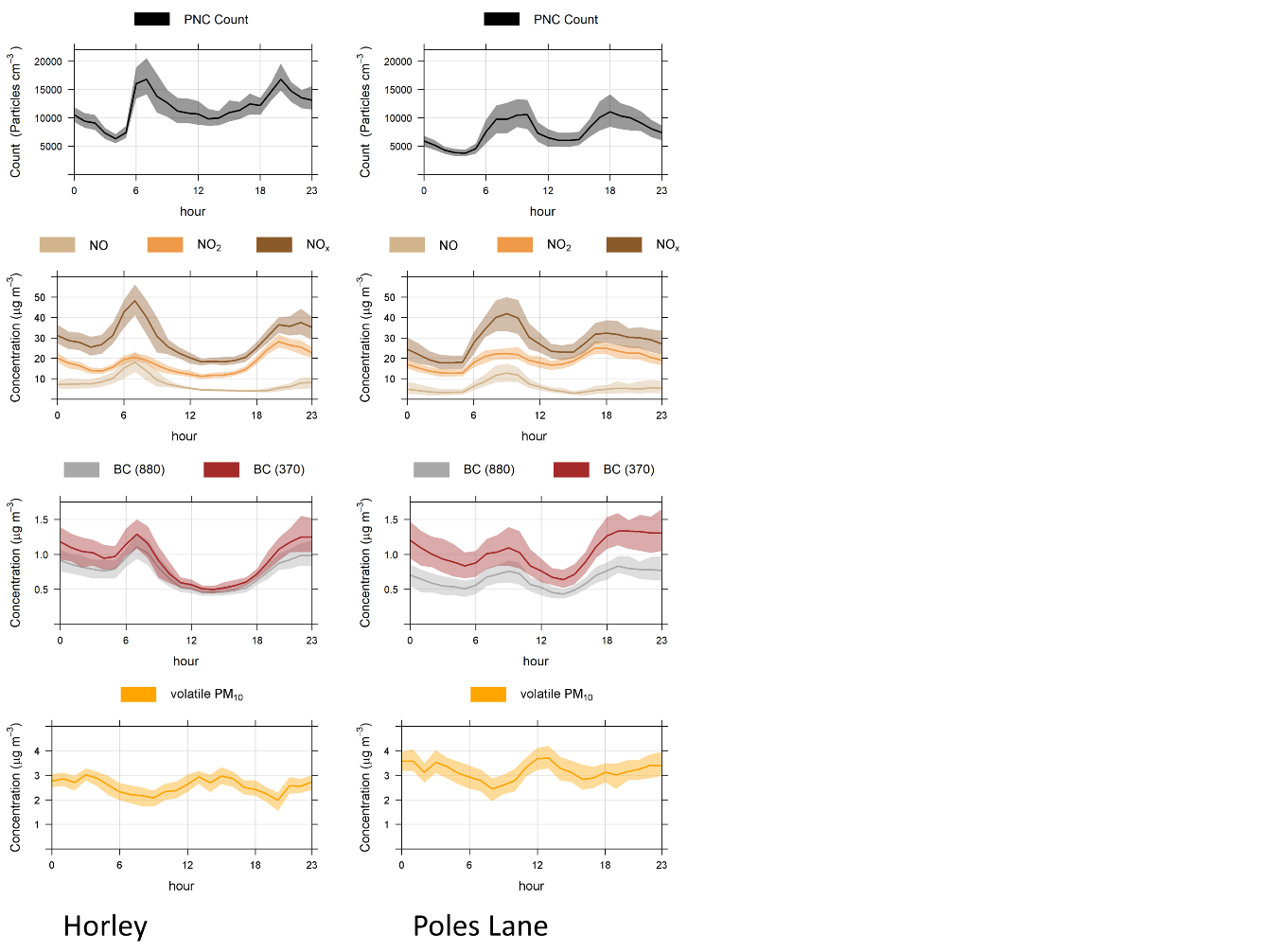


Figure S4: Diurnal variation of total particle number counts (PNC), NO/NO_2_/NO_X_ concentrations, BC(880)/BC(370) concentrations and volatile PM_10_ concentration in Horley (left) and Poles Lane (right)


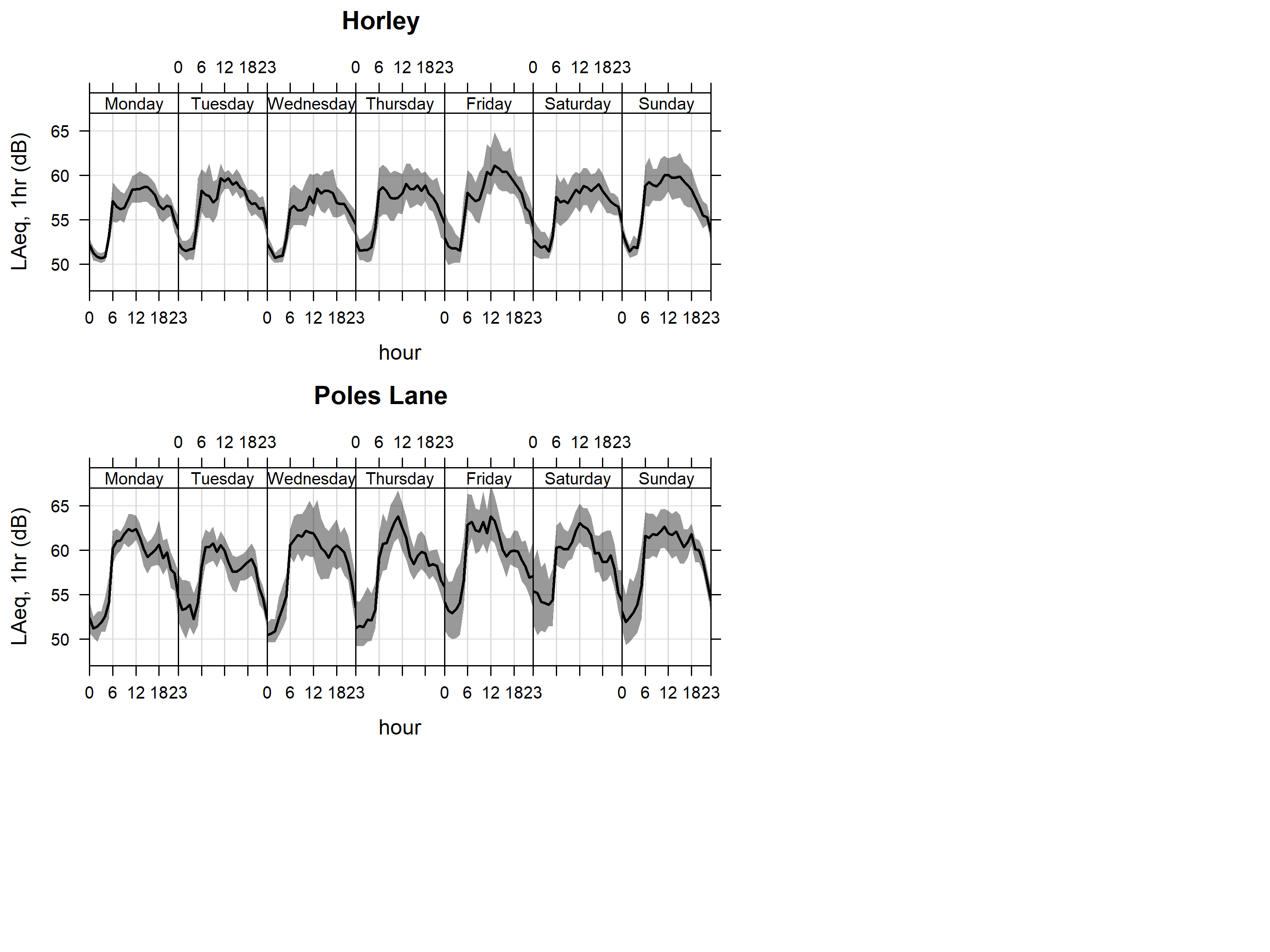


Figure S5: Combined hour of day/day of the week plots for noise levels in L_Aeq-1hr_ (dB) at Horley (top) and Poles Lane (bottom) during the respective study periods.


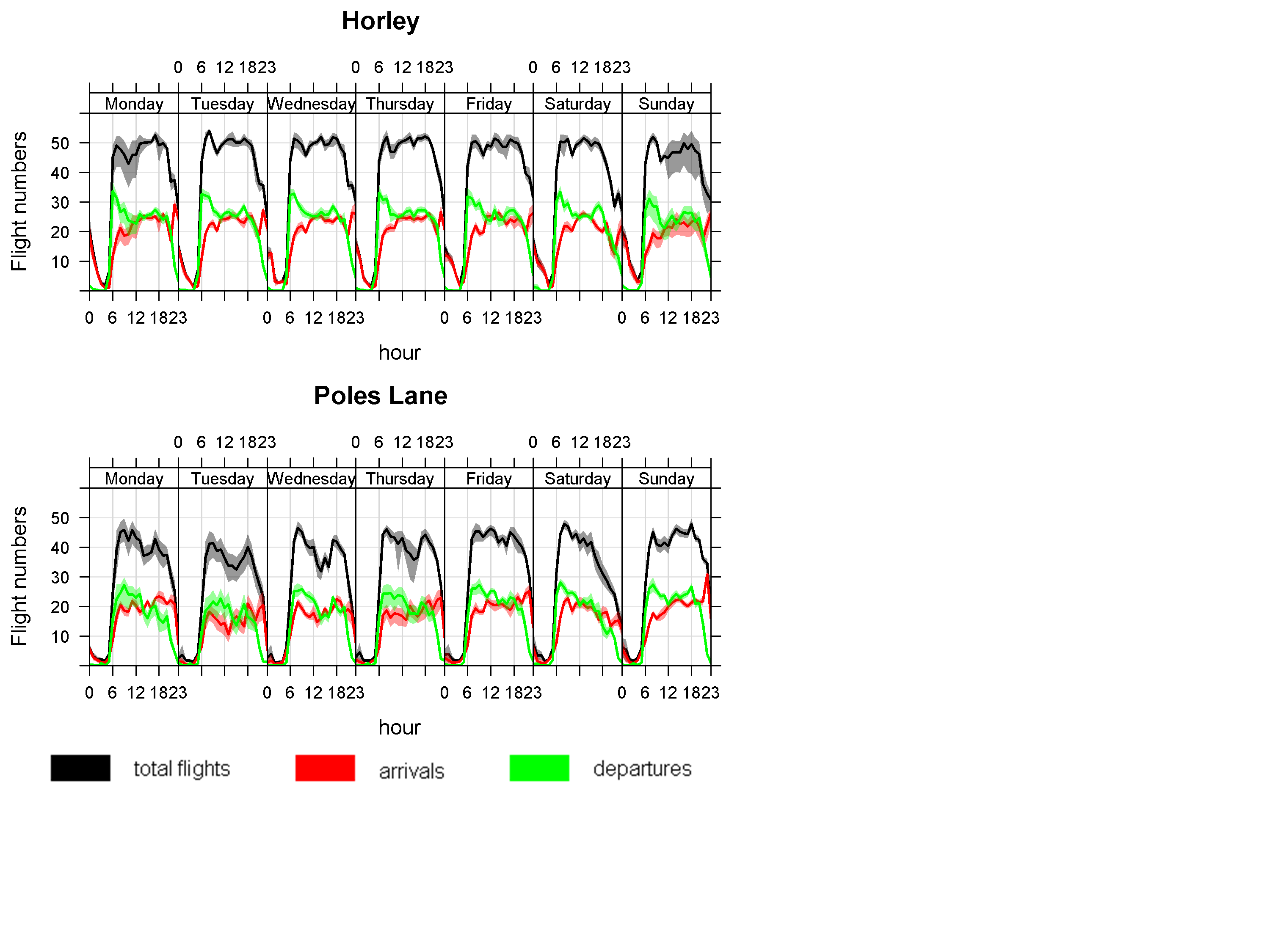


Figure S6: Combined hour of day/day of the week plots for the total flight numbers, as well as number of arrivals and departures at Horley (top) and Poles Lane (bottom) during the respective study periods.

**Supplement 2.2. Factors and Contributions**

To follow the guidance on reporting of PMF result given by (Brown et al. 2015), the following information is given in addition to what has been described in the method section of the main paper.

The model used for PMF, U.S. EPA PMF5, has a set lower limit of the normalised contribution of -0.2 to allow true rotations even if a large number of zero values are present. The model was run in robust mode. In this mode the influence of observation with scaled residuals greater than 4 are downweighed automatically. No constraints were used for the PMF analysis. The base model runs were carried out with 20 runs and seed number was set at 407.

The uncertainty of the input data was calculated following (Ogulei et al. 2007) with variations as used by (Rivas et al. 2020). Briefly, this approach uses an estimated measurement uncertainty, which was calculated using the following equation:

$\sigma_{ij}= \alpha_{j}*(N_{ij}+N_{j})$ (1)

with *σ_ij_* being the estimated measurement error for the size bin *j* and sample *i*; *N_ij_* is the observed concentration in size bin *j* and sample *i* and‾*N_j_* is the arithmetic mean of the observed concentration for the size bin j. *α_j_* is a constant for the size bin *j* and 0.025 was used in this study for both sites. Uncertainties have been increased in the lowest and highest size bins following the methodology of Rivas et al. (2020).

The overall uncertainty matrix was then calculated using *α_j_* (Eq. 1) as the estimated measurement error. The following formula was used:

$s_{ij}= \sigma_{ij}+C_{3}*N_{ij}$ (2)

C3 is an empirical constant, taken as 0.07 in this study for both sites.

The total PNC was not included in the model runs but the measured total PNC was used to obtain the source profiles and their contribution by regressing the modelled contribution matrix against the measured total PNC. As shown in Figure S8, the PNC predicted by PMF correlated well with the total measured PNC.

Displacement (DISP.) analysis was carried out to evaluate effects of rotational ambiguity. Rotational ambiguity refers to cases where the solution of the PMF algorithm is not unique *(*Comero et al. 2009). No factors swaps occurred in the DISP analysis for either site. For the dataset from Horley the largest observed drop in Q during the DISP analysis was -0.165; at Poles Lane the largest observed drop in Q was -0.205. The DISP intervals are given in the main paper text.

Bootstrap analysis (BS) evaluates the effects from random errors and partially of rotational ambiguity. Bootstrap analysis was carried out with 100 bootstrap runs with all other settings kept at default settings. In the main paper text, the 25^th^ and 75^th^ percentile were displayed as error limits in Figure 3. Table S1 shows the summary of bootstrap mapping. For the Horley data, all base and bootstrap factors were matched. For the Poles Lane data, the bootstrap factors matched the base case for all runs for factors 1 (urban), 5 (cooking) and 6 (aged traffic). Factor 3 (fresh traffic) was mapped to factor 6 (aged traffic) in one run and factor 4 (sec. aerosol A) was mapped to factor 1 (urban) twice and to factor 5 (cooking) twice. Factor 2 (airport) was mapped to the base case in 79 out of 100 runs. When factor 2 was mapped to other factors, these were mainly factors 1 and 6. The detection of this factor at Poles Lane was strongly dependent on the wind direction and with the site being upwind of the airport most of the time, the factor was not always identified in the BS.

Table S1: Mapping of Bootstrap factor to base factor for Horley and Poles Lane PMF analysis as given by US EPA PMF5

| **BS Mapping:** | **Horley** | | | | | | |
| --- | --- | --- | --- | --- | --- | --- | --- |
|  | Factor 1 | Factor 2 | Factor 3 | Factor 4 | Factor 5 | Factor 6 | Unmapped |
| **Boot Factor 1** | 100 | 0 | 0 | 0 | 0 | 0 | 0 |
| **Boot Factor 2** | 0 | 100 | 0 | 0 | 0 | 0 | 0 |
| **Boot Factor 3** | 0 | 0 | 100 | 0 | 0 | 0 | 0 |
| **Boot Factor 4** | 0 | 0 | 0 | 100 | 0 | 0 | 0 |
| **Boot Factor 5** | 0 | 0 | 0 | 0 | 100 | 0 | 0 |
| **Boot Factor 6** | 0 | 0 | 0 | 0 | 0 | 100 | 0 |
|  |  |  |  |  |  |  |  |
| **BS Mapping:** | **Poles Lane** | | | | | | |
|  | Factor 1 | Factor 2 | Factor 3 | Factor 4 | Factor 5 | Factor 6 | Unmapped |
| **Boot Factor 1** | 100 | 0 | 0 | 0 | 0 | 0 | 0 |
| **Boot Factor 2** | 8 | 79 | 1 | 2 | 2 | 8 | 0 |
| **Boot Factor 3** | 0 | 0 | 99 | 0 | 0 | 1 | 0 |
| **Boot Factor 4** | 2 | 0 | 0 | 96 | 2 | 0 | 0 |
| **Boot Factor 5** | 0 | 0 | 0 | 0 | 100 | 0 | 0 |
| **Boot Factor 6** | 0 | 0 | 0 | 0 | 0 | 100 | 0 |

The six-factor solution was chosen at each site as the ratio of Q(true) to Q(robust) (Figure S7) stepped closer to the ratio of 1 and the ratios levelled off with higher factor solutions. Q(true) is the goodness-of fit parameter, which includes all points in the model, whereas Q(robust) is the actual error function that is minimized by the model by excluding samples with uncertainty scaled residuals greater than 4. A higher factor solution was not considered a valid solution as a levelling off of the changes in the ratio of Q(true) to Q(robust) can be an indication of factor splitting (Brown et al., 2015). This six-factor solution also had the most physically meaningful profiles and temporal behaviour of the factors at both sites. The PMF predicted total UFP count correlated very well with the observed concentration for this solution at both sites and is given in Figure S8.


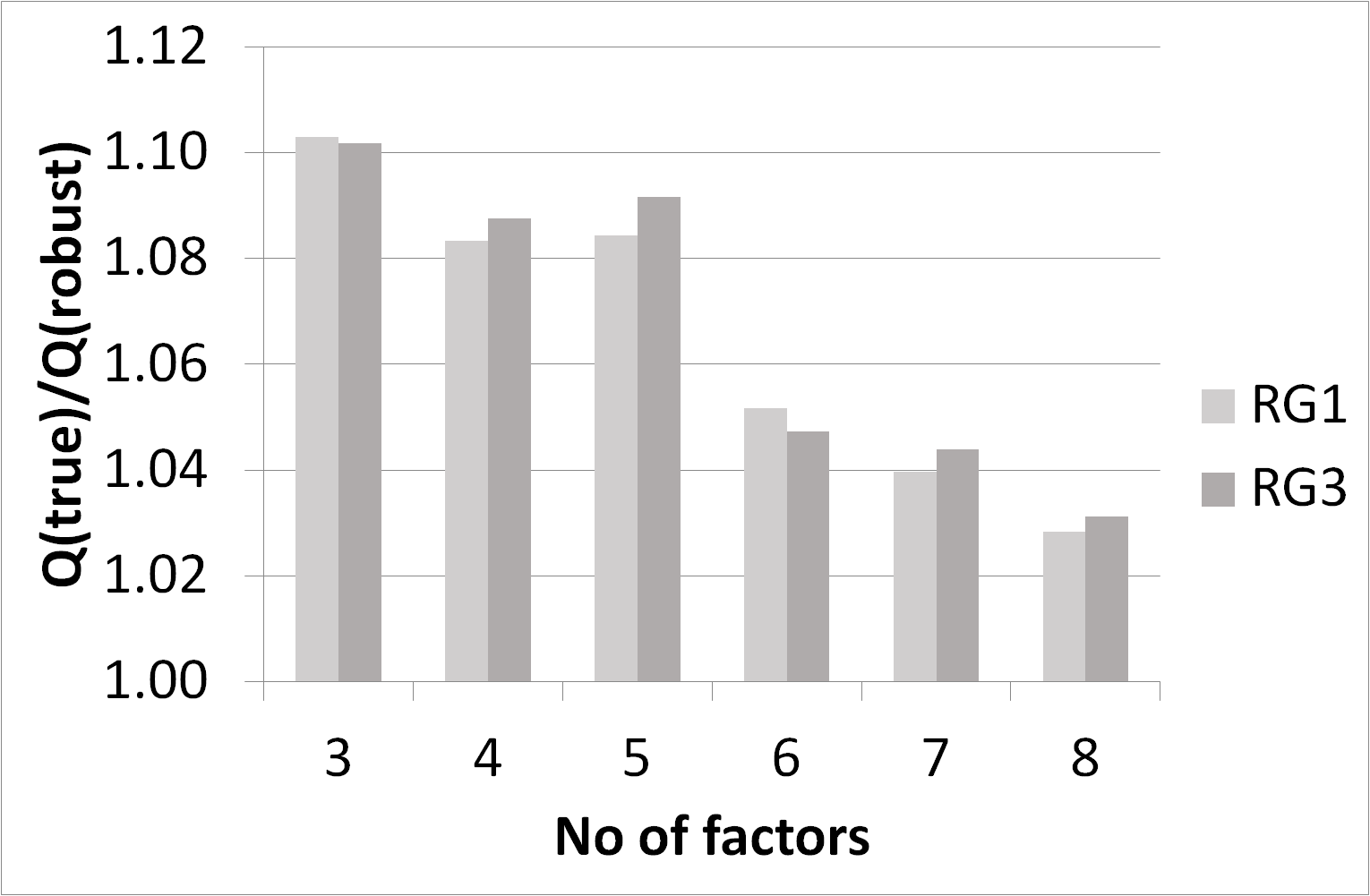


Figure S7: Q(true)/Q(robust) for Horley site (RG1) and Poles Lane site (RG3) for factor solutions 3 to 8


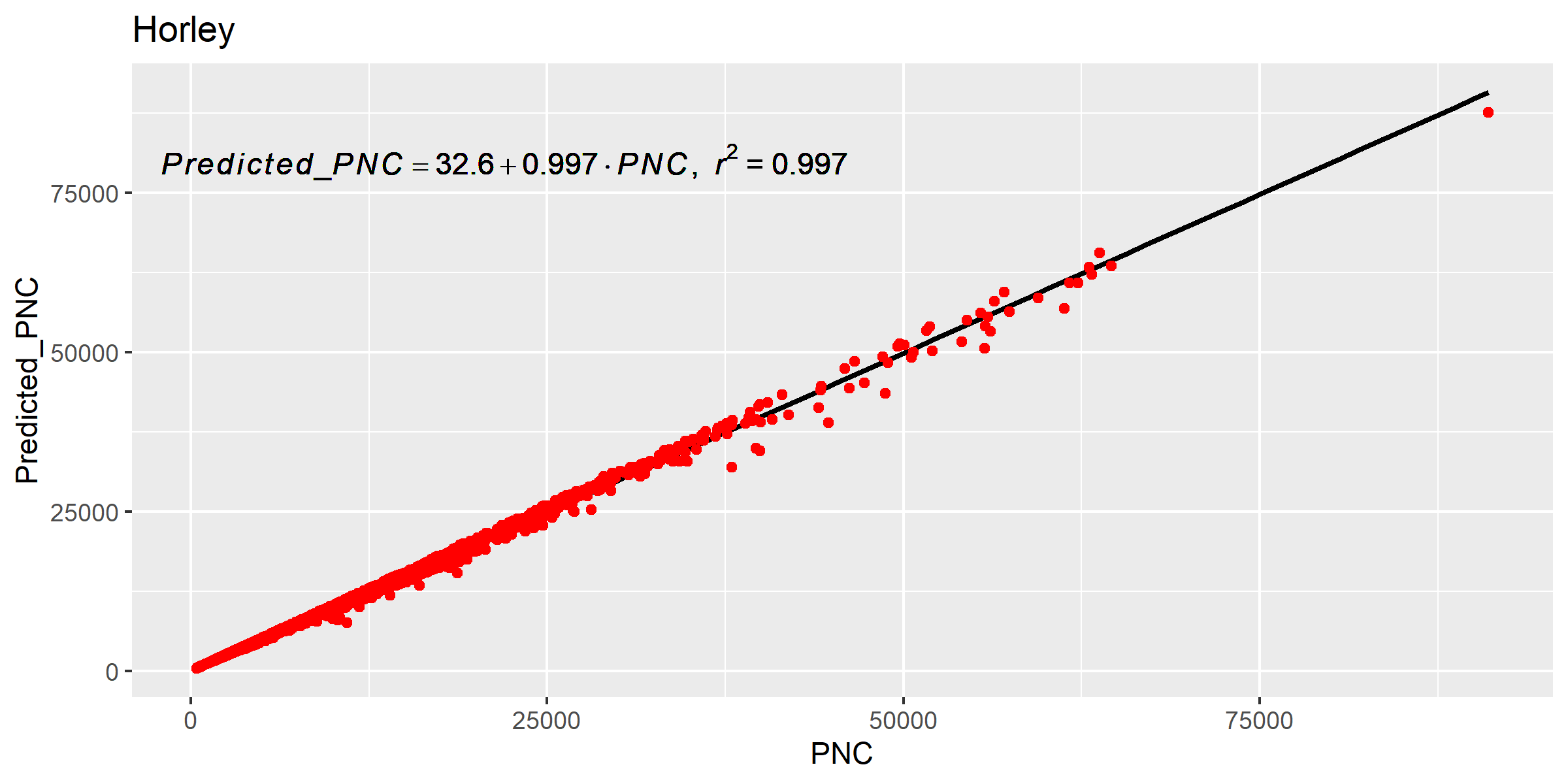

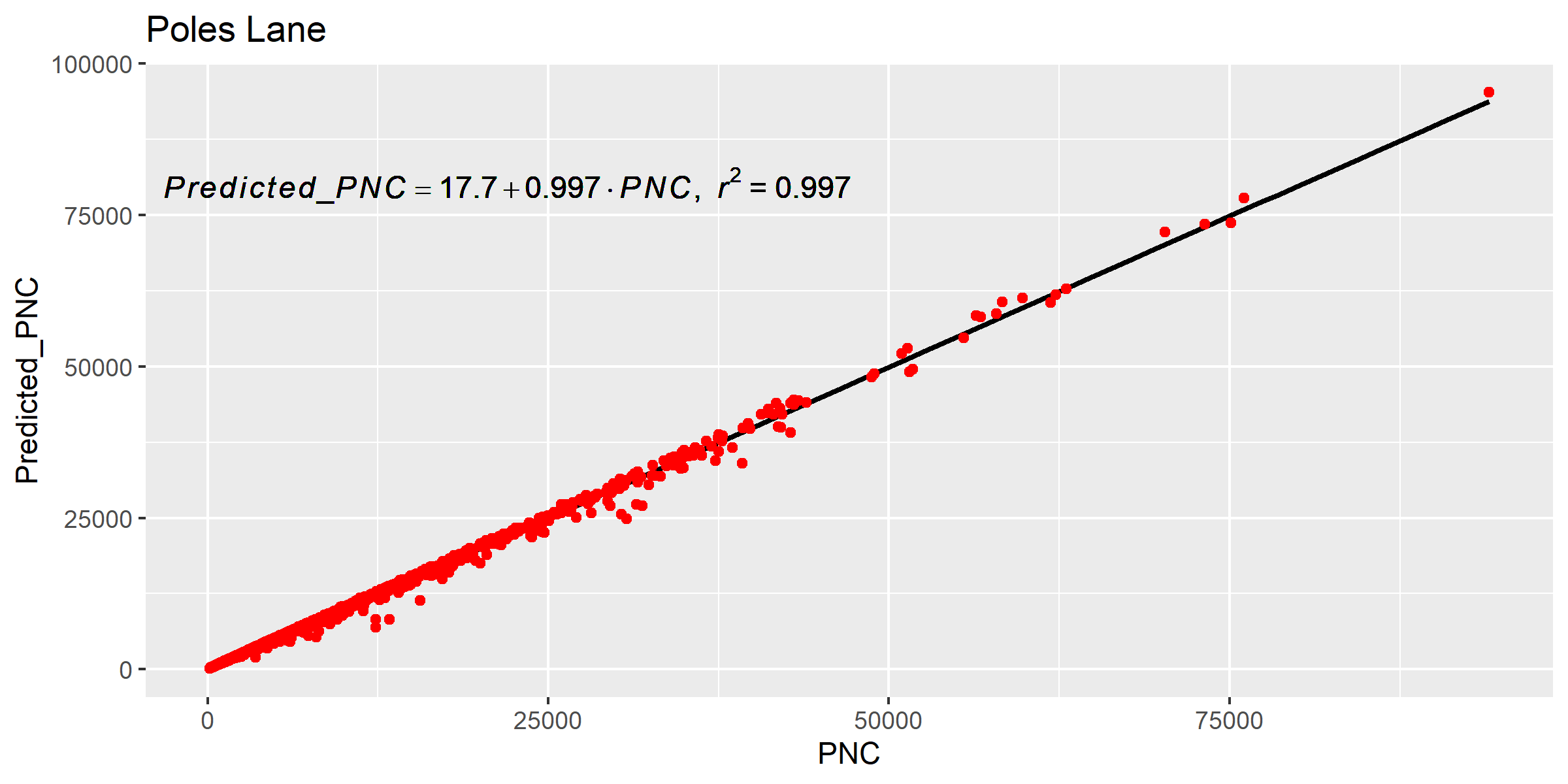


Figure S8: Correlation plots of observed particle number concentration (PNC) versus PMF predicted particle number concentration (Predicted_PNC) at Horley (top) and Poles Lane (bottom).

Table S2: Correlation Coefficient of factors and other pollutants At Horley and Poles Lane

|  | **Pearson's Correlation Coefficient for factors and pollutants at Horley** **Site** | | | | | |
| --- | --- | --- | --- | --- | --- | --- |
|  | **F1: airport** | **F2: fresh traffic** | **F3: aged traffic** | **F4: urban** | **F5: sec. aerosol A** | **F6: sec. aerosol B** |
| **F1: airport** | **1.00** |  |  |  |  |  |
| **F2: fresh traffic** | 0.49 | **1.00** |  |  |  |  |
| **F3: aged traffic** | 0.04 | 0.24 | **1.00** |  |  |  |
| **F4: urban** | -0.17 | -0.19 | 0.24 | **1.00** |  |  |
| **F5: sec. aerosol A** | -0.26 | -0.26 | 0.08 | **0.77** | **1.00** |  |
| **F6: sec. aerosol B** | -0.28 | -0.15 | -0.09 | 0.38 | 0.53 | **1.00** |
| **NO** | 0.00 | 0.11 | 0.42 | 0.52 | 0.37 | 0.14 |
| **NO_2_** | 0.29 | 0.57 | 0.43 | 0.34 | 0.24 | 0.13 |
| **NO_X_** | 0.14 | 0.35 | 0.52 | 0.55 | 0.39 | 0.17 |
| **BC_880_** | -0.07 | 0.11 | 0.43 | **0.77** | **0.64** | 0.42 |
| **BC_370_** | -0.09 | 0.05 | 0.39 | **0.83** | **0.70** | 0.43 |
| **PM_10VOL_** | -0.05 | -0.10 | -0.13 | 0.24 | 0.36 | 0.42 |
|  | **Pearson's Correlation Coefficient for factors and pollutants at Poles Lane Site** | | | | | |
|  | **F1: airport** | **F2: fresh traffic** | **F3: aged traffic** | **F4: urban** | **F5: sec. aerosol A** | **F6: cooking** |
| **F1: airport** | **1.00** |  |  |  |  |  |
| **F2: fresh traffic** | **0.68** | **1.00** |  |  |  |  |
| **F3: aged traffic** | 0.10 | 0.24 | **1.00** |  |  |  |
| **F4: urban** | -0.09 | 0.02 | 0.48 | **1.00** |  |  |
| **F5: sec. aerosol A** | -0.10 | -0.02 | 0.26 | **0.73** | **1.00** |  |
| **F6: cooking** | -0.02 | 0.00 | 0.18 | 0.37 | 0.39 | **1.00** |
| **NO** | 0.10 | 0.17 | 0.59 | **0.66** | 0.48 | 0.36 |
| **NO_2_** | 0.42 | 0.54 | 0.50 | 0.49 | 0.37 | 0.48 |
| **NO_X_** | 0.26 | 0.37 | **0.64** | **0.68** | 0.51 | 0.48 |
| **BC_880_** | 0.10 | 0.25 | **0.61** | **0.87** | **0.70** | **0.60** |
| **BC_370_** | 0.07 | 0.20 | 0.56 | **0.92** | **0.76** | **0.60** |
| **PM_10VOL_** | -0.07 | -0.05 | 0.13 | 0.40 | 0.53 | **0.67** |

**Supplement 2.4 Aircraft factor and noise**


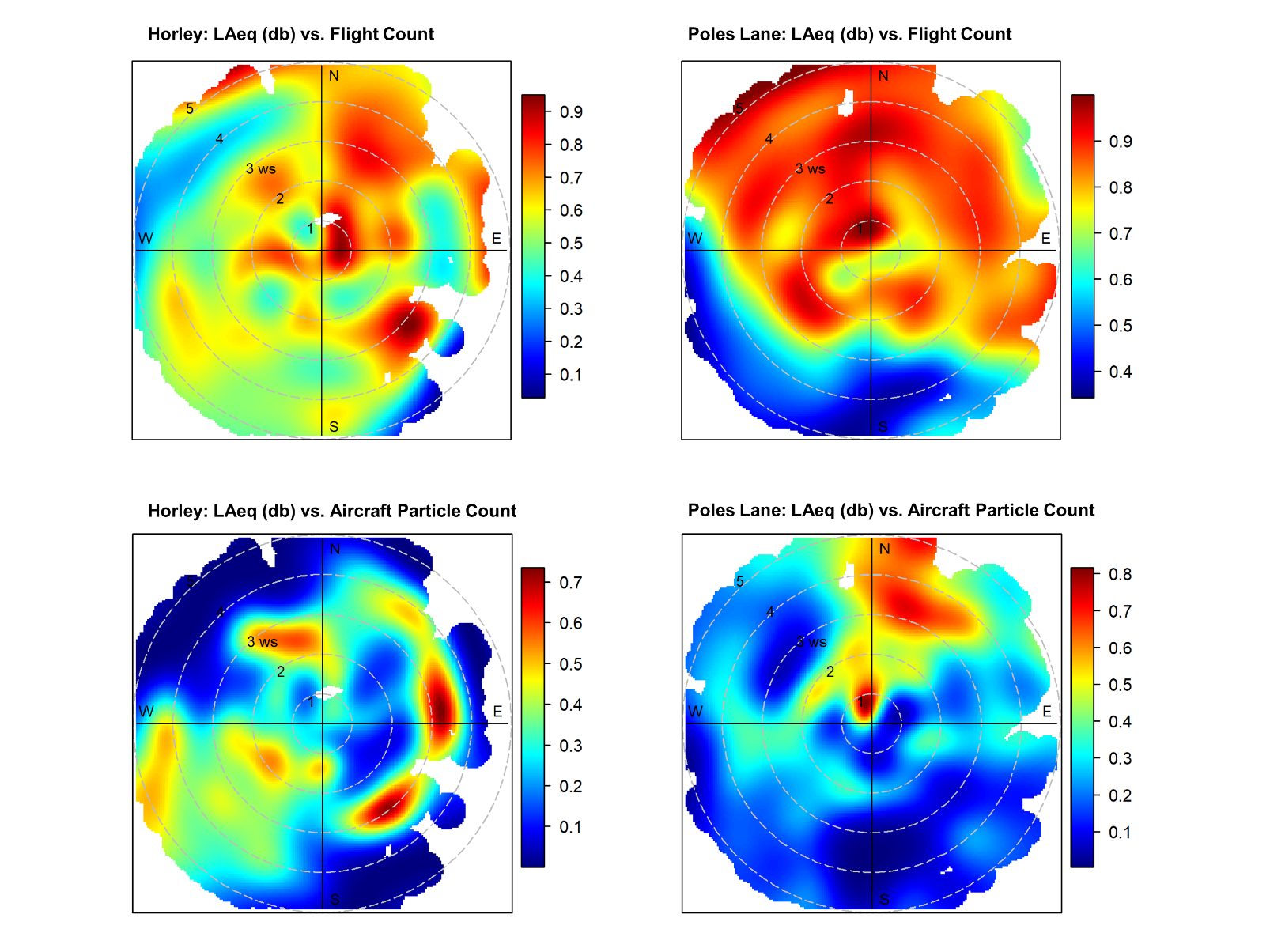


Figure S9: Bivariate polar plots illustrating the Pearson’s R correlation between L_Aeq-1hr_ sound levels and measurements of aircraft activity, by wind speeds of up to 5 m/s and wind direction.

The bivariate correlation between the noise measurements and the airport activity and modelled aircraft factor is shown in Figure S9. The correlations are shown for wind speeds up to 5 m/s as increased wind speed had a large influence on noise. To avoid extreme hours from masking a general trend 5 ms^-1^ was selected as a cut-off threshold because the 3rd quartile value at Horley = 4.35 ms^-1^ and Poles Lane = 4.85 ms^-1^ (i.e. 75% of the data is bellow this threshold).

The following general observations can be made:

- Horley Flight counts
  - The highest correlations are seen at wind speeds of <1 ms^-1^, indicating that a key source is in the general vicinity of the site
  - Another peak between noise and flight counts are associated with activity to the south-east of the site at wind speeds of 3 ms^-1^.
  - Moderate correlations generally observed in all directions
- Poles Lane Flight counts
  - High correlations between noise and flight activity (counts) are again observed at low wind-speeds.
  - The highest concentrations seem to arise from a major source to the north of the site.
  - The clear identification of the aircraft source reflects the sites proximity to the airport.
- Horley aircraft particle count
  - Moderate correlations are observed from a wide-spread source to the south-west of the site at wind speeds of 0-5 ms^-1^.
  - The highest correlations are observed at wind-speeds of 3 ms^-1^ from a source to the south-east and east of the site
- Poles Lane aircraft particle count
  - Correlation originates almost entirely from a single source to the north of the site.

Brown, S. G., S. Eberly, P. Paatero, and G. A. Norris. 2015. 'Methods for estimating uncertainty in PMF solutions: Examples with ambient air and water quality data and guidance on reporting PMF results', *Science of the Total Environment*, 518: 626-35.

Ogulei, D., P. K. Hopke, D. C. Chalupa, and M. J. Utell. 2007. 'Modeling source contributions to submicron particle number concentrations measured in Rochester, New York', *Aerosol Science and Technology*, 41: 179-201.

Rivas, I., D. C. S. Beddows, F. Amato, D. C. Green, L. Jarvi, C. Hueglin, C. Reche, H. Timonen, G. W. Fuller, J. V. Niemi, N. Perez, M. Aurela, P. K. Hopke, A. Alastuey, M. Kulmala, R. M. Harrison, X. Querol, and F. J. Kelly. 2020. 'Source apportionment of particle number size distribution in urban background and traffic stations in four European cities', *Environment International*, 135.

Comero S, Capitani L, Gawlik B. 2009. 'Positive Matrix Factorisation (PMF) - An Introduction to the Chemometric Evaluation of Environmental Monitoring Data Using PMF', EUR 23946 EN. Luxembourg (Luxembourg): OP; 2009. JRC52754
